# Monovalent and unpoised status of most genes in undifferentiated cell-enriched *Drosophila *testis

**DOI:** 10.1186/gb-2010-11-4-r42

**Published:** 2010-04-15

**Authors:** Qiang Gan, Dustin E Schones, Suk Ho Eun, Gang Wei, Kairong Cui, Keji Zhao, Xin Chen

**Affiliations:** 1 Department of Biology, The Johns Hopkins University, 3400 North Charles Street, Baltimore, MD 21218, USA; 2 Laboratory of Molecular Immunology, National Heart, Lung and Blood Institute, National Institutes of Health, 10 Center Drive, Building 10, Bethesda, MD 20892, USA

## Abstract

**Background:**

Increasing evidence demonstrates that stem cells maintain their identities by a unique transcription network and chromatin structure. Opposing epigenetic modifications H3K27me3 and H3K4me3 have been proposed to label differentiation-associated genes in stem cells, progenitor and precursor cells. In addition, many differentiation-associated genes are maintained at a poised status by recruitment of the initiative RNA Polymerase II (Pol II) at their promoter regions, in preparation for lineage-specific expression upon differentiation. Previous studies have been performed using cultured mammalian embryonic stem cells. To a lesser extent, chromatin structure has been delineated in other model organisms, such as *Drosophila*, to open new avenues for genetic analyses.

**Results:**

Here we use testes isolated from a *Drosophila bag of marbles *mutant strain, from which germ cells are in their undifferentiated status. We use these testes to study the endogenous chromatin structure of undifferentiated cells using ChIP-seq. We integrate the ChIP-seq with RNA-seq data, which measures the digital transcriptome. Our genome-wide analyses indicate that most differentiation-associated genes in undifferentiated cells lack an active chromatin mark and initiative Pol II; instead, they are associated with either the repressive H3K27me3 mark or no detectable mark.

**Conclusions:**

Our results reveal that most of the differentiation-associated genes in undifferentiated-cell-enriched *Drosophila *testes are associated with monovalent but not bivalent modifications, a chromatin signature that is distinct from the data reported in mammalian stem or precursor cells, which may reflect cell type specificity, species specificity, or both.

## Abstract 2

In undifferentiated Drosophila cells, differentiation-associated genes have monovalent, not bivalent histone modifications, in contrast to differentiation-associated genes in stem cells.

## Background

Extensive studies indicate that embryonic stem cells (ESCs), lineage-committed adult stem cells and early progenitor cells maintain their identities by a unique transcriptional network and chromatin structure (reviewed by [1,2]). In particular, the bivalent domains harboring both the active H3K4me3 and repressive H3K27me3 marks label developmental regulators [3]. The H3K4me3 and H3K27me3 marks are placed by the Trithorax group (TrxG) complex [4-6] and the Polycomb group (PcG) complex [7,8], respectively. Increasing evidence indicates that the PcG and the TrxG complexes play critical roles in the choice between the proliferating progenitor cell state and terminal differentiation program [4,9]. It has been reported that bivalent genes in ESCs or early progenitor cells are bound by PcG proteins and are maintained at a 'poised' status by recruitment of RNA Polymerase II (Pol II), in preparation for lineage-specific expression upon differentiation [10-12]. In various stem cell lineages, reversal of repression by the PcG silencing machinery may act as the first step toward robust activation of terminal differentiation genes [13,14].

The *Drosophila *male germline stem cell (GSC) lineage is a naturally existing adult stem cell system and has emerged as an excellent system for studying the molecular mechanisms that control stem cell maintenance versus differentiation [15]. Each GSC divides asymmetrically to self-renew and give rise to a gonialblast, the daughter cell that first undergoes a transit-amplifying spermatogonial stage before converting to differentiating spermatocytes [16]. The maintenance of GSCs and spermatogonia in an undifferentiated and proliferative state, as well as the subsequent reversal of these controls to allow terminal differentiation, are both critical to continuous production of gametes throughout lifetime. Despite extensive genetic studies on maintenance of GSCs, it is unclear how epigenetic mechanisms may establish and maintain a unique chromatin structure for their undifferentiated status; and how mis-regulation of such a structure may lead to mis-determination of their fate [14].

Previous studies in this system have shown that PcG transcriptional silencing proteins repress the genes required for terminal differentiation in undifferentiated germ cells. Developmental programs reverse Polycomb silencing and activate the expression of differentiation genes in spermatocytes [17]. This work uncovered an intriguing parallel between *Drosophila *GSC and ESC lineages, because PcG proteins play an extensive role in keeping developmental regulators at a silent status in murine and human ESCs [10,11]. To investigate whether other features in mammalian ESCs apply to *Drosophila*, we studied the chromatin structure in the undifferentiated-cell-enriched *Drosophila *testis. Our results revealed two distinct features in this tissue: a monovalent chromatin signature and lack of paused RNA Pol II at the differentiation genes. Both features are different from what have been reported for ESCs and other mammalian adult stem cells, suggesting a potential novel mechanism of regulating the germ cell differentiation program in *Drosophila *testis.

## Results and discussion

### Summary of the ChIP-seq results in undifferentiated-cell-enriched *Drosophila *testis

Since it is unfeasible to obtain a sufficient number of naturally existing GSCs for epigenomic mapping using current chromatin immunoprecipitation (ChIP) techniques, we took advantage of the *bag of marbles *(*bam*) mutant strain. Mutations in the *bam *gene inhibit the transition from spermatogonial progenitor stage to differentiating spermatocytes, which results in an accumulation of undifferentiated cells, including GSCs, transit-amplifying spermatogonial cells, as well as somatic cells [18,19]. Although the *bam *testes are not a pure source of GSCs, they are enriched with undifferentiated germ cells and have been used for transcription profiling by microarray [20] or RNA-seq [21] to search for undifferentiated-cell-enriched genes. These analyses further confirmed the undifferentiated status of cells in *bam *testes, in which the expression of meiotic and terminal differentiation genes was extremely low or undetectable.

To investigate the epigenetic mechanisms that regulate the male germline cellular differentiation program, we used *bam *testes for chromatin landscape mapping using ChIP followed by high-throughput sequencing (ChIP-seq). The ChIP-seq technique has been demonstrated to be a highly sensitive method to detect binding sites of chromatin-associated proteins at a genome-wide coverage (reviewed by [22,23]). More importantly, this technique is compatible with small amounts of starting material, such as the hand-dissected fly testes in our experiments. To compare our data with the previous results using mammalian cells, we used the same set of antibodies, including antibodies against RNA Pol II, H3K4me3, H3K36me3, H3K27me3 and unmodified histone H3, to perform ChIP-seq experiments using *bam *testes (Materials and methods; Additional file 1). The anti-Pol II antibody (4H8) used in our assays recognizes both the initiating and elongating forms of Pol II according to published work [12,24]. Sequencing depth analysis indicated that we have reached or almost reached the plateau of peak detection for each of these histone modifications as well as Pol II (Materials and methods; Additional file 2a-d).

**Figure 1:**
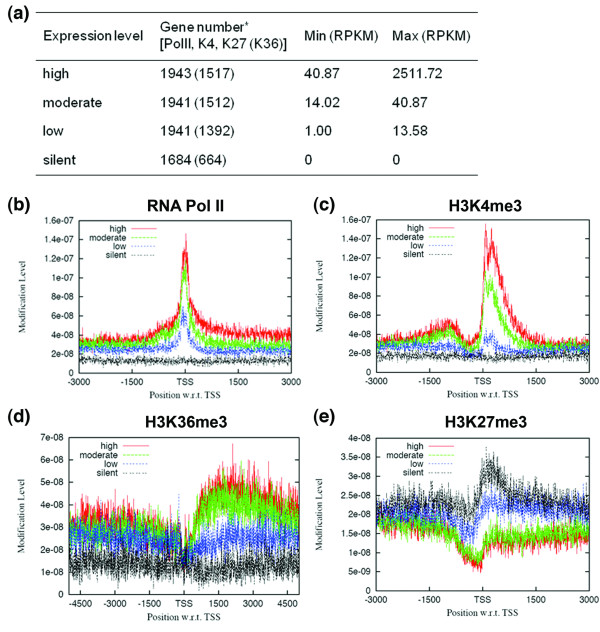
**Summary of the ChIP-seq results using *bam *testis**. **(a) **The four groups of genes were classified according to their RPKM value based on the RNA-seq results [21]. The numbers in brackets denote genes used for H3K36me3 (K36) analysis. *See Materials and methods for gene selection criteria. Antibodies used for ChIP-seq were: **(b) **anti-RNA Pol II (Pol II); **(c) **anti-H3K4me3 (K4); **(d) **anti-H3K36me3 (K36); and **(e) **anti-H3K27me3 (K27). Enrichment of each histone modification and RNA Pol II is plotted over a -3-kb to +3-kb region with respect (w.r.t.) to genes' TSSs, except for K36, for which a -5-kb to +5-kb region is used.

To validate our ChIP-seq data, we compared the relationship between gene expression level and enrichment of RNA Pol II as well as distinct histone modifications. The *Drosophila *genome is highly compact, including many overlapping genes and genes with transcriptional start sites (TSSs) within a short distance of one another, complicating interpretations of ChIP-seq results. To avoid this, we classified 9,459 annotated genes that are applicable for ChIP-seq analysis into four groups (Materials and methods) according to their expression levels determined by RNA-seq in RPKM values [21,25,26] (Figure 1a; RPKM is sequencing reads per kilobase of exon per million mapped reads [26]). Our data demonstrated that both RNA Pol II and H3K4me3 were highly elevated near the TSSs of annotated genes. They also positively correlated with gene expression level (Figure 1b, c). And H3K36me3 was enriched downstream of the TSSs and also positively correlated with expression level (Figure 1d). In contrast, H3K27me3 was negatively correlated with gene expression, as expected for a repressive mark (Figure 1e). These results are consistent with observations in human T cells [27] and mammalian ESCs [28].

### Monovalent chromatin signature is prevalent in undifferentiated-cell-enriched *bam *testis

The coexistence of active H3K4me3 and repressive H3K27me3 marks is detected in a large number of developmental regulator genes in mammalian ESCs [3,29,30] and adult stem/progenitor cells [31,32]. These 'bivalent' modifications are proposed to poise genes for rapid induction during cellular differentiation to become different cell lineages. However, the mechanisms that contribute to the bivalency of genes may be diverse under different circumstances, and still remain unsolved at the structural level [33].

**Figure 2:**
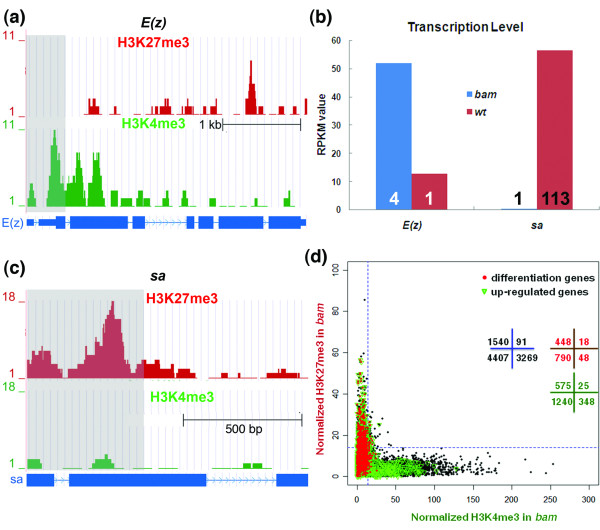
**Monovalent chromatin signature is prevalent in undifferentiated cells of *bam *testis**. **(a) **UCSC genome browser screenshot showing the H3K4me3 monovalency at the *E(z) *gene locus in undifferentiated cells. The read counts are labeled on the y-axis and the genomic region used to calculate the enrichment of modified histones (0 to +500 bp with respect to the TSS) is shaded in grey. **(b) **Transcription level of two representative monovalent genes, *E(z) *and *sa*, in *bam *and *wild-type *(*wt*) testis, respectively. According to the RNA-seq data, the RPKM for *E(z) *mRNA is four-fold higher in *bam *testis, and that for *sa *mRNA is 113-fold higher in wild-typetestis. **(c) **UCSC genome browser snapshot showing the H3K27me3 monovalency at the *sa *gene locus in undifferentiated cells. The read counts are labeled on the y-axis and the genomic region used to calculate the enrichment of modified histones (0 to +500 bp with respect to the TSS) is shaded in grey. **(d) **Scatter plot for H3K4me3 and H3K27me3 enrichment of all annotated genes. A common window (0 to +500 bp with respect to the TSS) was used to calculate H3K4me3 and H3K27me3 reads. The blue dashed lines indicate the statistical cutoff line - *P *< 0.05 in a 1-kb window per 1 million total reads. Gene numbers in each quadrant are labeled in black. Differentiation genes are defined as those with RPKM in *bam *testis < 0.5 and RPKM in wild-type testis ≥ 1 and are labeled as red dots. Among the 1,894 differentiation genes, 1,304 genes are applicable for ChIP-seq analysis (Materials and methods). Up-regulated genes are defined as those with RPKM in wild-typetestis/*bam *testis ≥ 2 (if the RPKM in *bam *testis < 0.5, the value is raised to 0.5) and are labeled as green inverted triangles. Among the 3,377 up-regulated genes, 2,188 genes are applicable for ChIP-seq analysis (Materials and methods).

To test whether bivalency also applies to the undifferentiated cells of *Drosophila *testis, we first examined several critical genes that regulate cellular differentiation in the GSC lineage. The *Enhancer of Zeste *(*E(z)*) gene encodes a PcG complex component that is highly expressed in undifferentiated cells, but abruptly down-regulated upon differentiation initiation in spermatocytes. The co-existence of H3K27me3 and H3K4me3 was not observed at the *E(z) *gene locus near the TSS. Instead, we found only the active H3K4me3 mark at the *E(z) *gene locus, enriched near the TSS (Materials and methods; Figure 2a, b). The *spermatocyte arrest *(*sa*) gene, which encodes a testis-specific homolog of TBP associated factor (tTAF), is turned on in spermatocytes. The *tTAF *gene acts to antagonize PcG repression and regulate a cell-type-specific transcription program for terminal differentiation [17,34] (Figure 2b). In contrast to the *E(z) *gene, we found that the chromatin of the *sa *gene locus was only associated with the repressive H3K27me3 mark near its TSS in *bam *testis (Figure 2c). More examples of the histone modification patterns of differentially expressed genes are shown in Additional file 3. These data suggest that developmental regulator genes in the germline lineage may be associated with a 'monovalent' modification (either H3K4me3 or H3K27me3 mark) in undifferentiated cells of *bam *testis.

To test the prevalence of monovalency at a genome-wide scale, we analyzed all 9,459 annotated genes that are applicable for ChIP-seq analysis (Materials and methods). We identified 3,360 genes that were enriched with a significant level of active H3K4me3 and 1,631 genes enriched significantly with repressive H3K27me3 (*P *< 0.05; Materials and methods). Surprisingly, only 91 genes were associated with both H3K4me3 and H3K27me3 marks (Figure 2d).

Although we could validate several bivalent genes using independent ChIP followed by real-time PCR analysis (Additional file 4), we found that, different from the silent status of bivalent genes in ESCs [3], 56% of the bivalent genes in *bam *testis were actively expressed (Additional file 5; the RPKM cutoff for expressed genes is based on Additional file 6), indicating that the bivalency may be a result of temporally and/or spatially regulated gene expression, as has been reported in *Xenopus *embryos [35,36]. One particular example is the *benign gonial cell neoplasm *(*bgcn*) gene (Additional file 4b), whose product acts with the Bam protein in the transition of germ cells from the proliferative to the differentiating stage [18]. We reasoned that the bivalency at the *bgcn *gene may have resulted from mixed cell types in *bam *testes. The active status of *bgcn *in spermatogonial cells is likely associated with the enriched active H3K4me3 mark, whereas its repressive status in GSCs and somatic cells may contribute to the detection of the repressive H3K27me3 mark. Consistent with this, we found that none of the 91 putative bivalent genes we identified in *bam *testis retained their bivalency in the cultured *Drosophila *cells, including S2, BG3 and D23 cell lines [37] (Additional files 5 and 7; Materials and methods). Although the culture cells were not synchronized, they were more pure than tissues with mixed types and staged cells. In addition, we performed Gene Ontology analysis to search for significantly enriched gene categories in both molecular function and biological process terms (Materials and methods). Surprisingly the only category that is significantly enriched (*P *< 0.01) for these 91 putative bivalent genes is the 'multicellular organismal process' category, further suggesting that the apparent bivalency may come from different cells and is not specifically involved in cellular differentiation during spermatogenesis.

To test the prevalence of monovalency for all differentiation genes in testis, we compared the expression profile of fully differentiated wild-type testis with undifferentiated *bam *testis using the RNA-seq data [21]. We found 1,894 genes that were silent in the undifferentiated-cell-enriched *bam *testis (RPKM < 0.5) but were turned on in the differentiated cells from wild-type testis (RPKM ≥ 1). We defined these genes as differentiation genes (the RPKM cutoffs are based on Additional file 6). Among these 1,894 differentiation genes, 1,304 are applicable for ChIP-seq analysis (Materials and methods). Examination of the histone modifications on these 1,304 genes revealed that only 18 genes were associated with both active H3K4me3 and repressive H3K27me3 in *bam *testis (Figure 2d; Additional file 5), whereas 448 were associated with only H3K27me3, 48 were associated with only H4K4me3 and 790 were not associated with either of these modifications. We also checked all genes that are up-regulated at least two-fold in wild-type testis, regardless of their expression level in *bam *testis (RPKM in wild-type testis/*bam *testis ≥ 2). We found 3,377 genes that fall into this category, including all differentiation genes. Among these 3,377 up-regulated genes, 2,188 are applicable for ChIP-seq analysis. We analyzed the H3K27me3 and H3K4me3 enrichment of these genes and found only 25 genes enriched with both histone marks (Figure 2d). These results indicate that most differentiation genes in undifferentiated-cell-enriched testis are marked by either a monovalent chromatin signature or no modification (H3K4me3 or H3K27me3), which is very different from the modification patterns in mammalian ESCs [3] or progenitor cells [32]. Consistent with our findings, previous studies demonstrated the paucity of bivalent domains in fly embryos, which contain progenitor cells mainly for somatic tissues [38]. In summary, our results reveal that monovalent modification is a prevalent chromatin signature of differentiation genes in undifferentiated-cell-enriched *Drosophila *testis.

### Most genes are unpoised in the undifferentiated-cell-enriched *bam *testis

**Figure 3:**
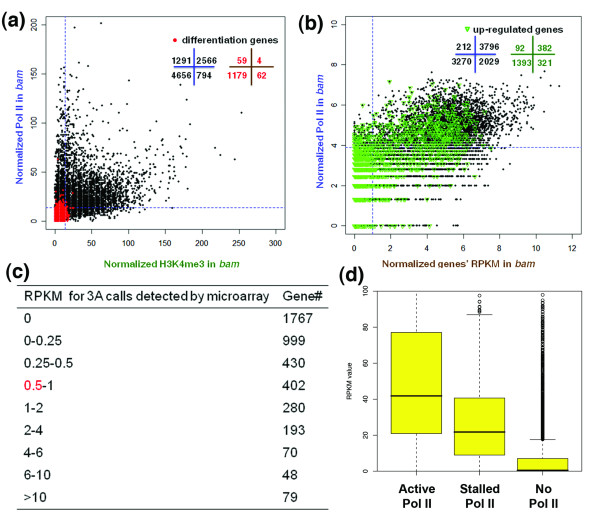
**Most genes are unpoised in undifferentiated cells of *bam *testis**. **(a) **Scatter plot for H3K4me3 and RNA Pol II enrichment of all annotated genes. All labels and the definition of up-regulated genes are the same as in Figure 2d, except that a -250 to +250-bp window with respect to the TSS was used to calculate reads for RNA Pol II. **(b) **Scatter plot for RNA Pol II enrichment and RPKM values for all annotated genes. All labels are the same as in Figure 2d, except that RPKM ≥ 1 is used as a cutoff for expressed genes. **(c) **RPKM values for genes that have three absent calls (3A) in the microarray. **(d) **Analysis of the RPKM values of transcripts demonstrates that genes with stalled Pol II are weakly transcribed genes. Genes that show an active Pol II profile are expressed at significantly higher levels. Genes with no Pol II profile are expressed at significantly lower levels. The box represents the 25th and 75th percentiles, with the 50th percentile as a black bar. The whiskers refer to outliers that are at least 1.5× the interquartile range from the box. The y-axis represents the RPKM value.

For the purpose of discussion here, we use 'poised' to describe the prepared status of a gene for transcription [3,24,39-42], which is associated with the promoter proximal binding of RNA Pol II at a 'paused' status [43-49] and/or with active histone modification marks [12,32]. Previous studies in mammalian ESCs [12] and *Drosophila *embryos [39] have suggested that many differentiation genes are unexpressed yet have paused RNA Pol II associated with their promoters, in order to stay at a poised state ready for robust transcription upon developmental stimuli. For example, more than 13% of the ectodermal differentiation genes were repressed but have paused RNA Pol II in the *Toll*^*10b *^mutant *Drosophila *embryos, in which all cells are transformed to the mesodermal fate [39]. Here we examined whether paused Pol II is also prevalent in the undifferentiated-cell-enriched male gonads. Surprisingly, we found only 63 (4.8%) of the 1,304 differentiation genes were bound by Pol II (*P *< 0.05) in undifferentiated-cell-enriched *bam *testis (Figure 3a). These data indicate that most genes that are silent in undifferentiated cells (RPKM in *bam *testis <0.5) but turned on upon differentiation (RPKM in wild-type testis ≥ 1) remained at an unpoised status in undifferentiated cells.

On the other hand, analyses of the expression levels of all genes with significant Pol II binding (*P *< 0.05) demonstrated that approximately 95% of genes (3,796 genes) that are enriched with Pol II (4,008 genes) were actively expressed (RPKM in *bam *testis ≥ 1; Figure 3b). Only 5% of Pol II-enriched genes (212 genes) were expressed at very low to undetectable levels (RPKM <1), which may be candidate poised genes in this cell lineage but only comprised 2% of all genes analyzed (9,459 genes). Interestingly, we found that a subset of these potentially poised genes (43%; 92 genes in Additional file 4b) are up-regulated upon differentiation (RPKM in wild-type testis/*bam *testis ≥ 2), with the significantly (*P *< 0.01) enriched ontology category 'transcription regulator activity'. In contrast, 75 genes (35%) remain silent throughout spermatogenesis (RPKM <0.5 in both wild-type and *bam *testis). Consistent with their low or undetectable expression (RPKM <1) in *bam *testis, we found that only 28 out of the 212 (13.2%) potentially poised genes are enriched with H3K36me3, a transcription elongation epigenetic mark [27]. Furthermore, we compared these 92 putative poised genes with the 91 potential bivalent genes in *bam *testis. Surprisingly, only two genes (*CG8517 *and *CG13611*) overlapped. These data demonstrate that different from what has been reported in mammalian ESC lineages [10-12], most differentiation genes stay at an unpoised status before exposure to the developmental signals in the undifferentiated-cell-enriched *Drosophila bam *testis.

In addition to RNA Pol II, H3K4me3 is another well-established mark for active chromatin status, and is associated with many unexpressed but poised genes in ESCs [12]. In our analysis, we found 95.4% of all H3K4me3-enriched genes (3,360 genes) are actively expressed (3,204 genes in Additional file 8). This result further confirmed that most genes in undifferentiated-cell-enriched *Drosophila *testis are either actively expressed or silent, without an intermediate poised status.

### Direct comparison of RNA-seq and microarray data reveals genes with low expression levels may contribute to the paused Pol II phenomenon

One explanation for the difference in our results from previous publications is that previous analysis of gene expression mainly relied on microarray studies, which may not be sensitive enough to distinguish genes with low expression levels from genes that are absolutely silent [50]. Indeed, when we compared the gene expression profiles in *bam *testis determined by microarray versus RNA-seq techniques, we found that 670 genes considered as silent based on microarray analysis (three 'absent' calls in all three biological replicates) were actually expressed according to their RPKM values in the RNA-seq data set (RPKM ≥ 1; Figure 3c). Approximately 24.8% of these genes (107 of the 432 genes that are applicable for ChIP-seq analysis among all 670 genes) had significant enrichment of Pol II (*P *< 0.05) at their promoter regions. Therefore, our results suggest that some of the 'poised' genes may actually be expressed but below the detection threshold in microarray analysis.

Since the Pol II antibody we used in our ChIP-seq experiment recognizes both the initiating and elongating (Ser5 phosphorylated) forms [12,24], we used the Pol II ChIP-seq data to calculate the stalling index for each gene ([39] and Materials and methods). Using this assay, we identified 695 genes that have paused Pol II at their promoter regions in *bam *testis (Additional file 9a; Materials and methods). Ontology analysis revealed significant functional categories (*P *< 0.01) for these genes (Additional file 9b). However, approximately 93.0% (647 out of 695) of these genes were expressed (RPKM ≥ 1), albeit at relatively low levels (Figure 3d).

### Do different techniques contribute to the different features of the chromatin landscape?

There are two major differences between our work and published studies in *Drosophila *or mammalian cells: first, we used direct sequencing-based techniques (ChIP-seq and RNA-seq) instead of hybridization-based techniques (ChIP-chip and microarray); and second, we used a tissue that is enriched with undifferentiated germ cells instead of embryos [39], embryo-derived cell lines [51], or mammalian cells [12], which mainly comprise cells with somatic fate. In order to make a more direct comparison to address whether the difference is due to distinct techniques or different cell types, we performed ChIP-seq and RNA-seq analyses using the same cell type - *Drosophila *S2 cells - that was used in a previous study [51].

**Figure 4:**
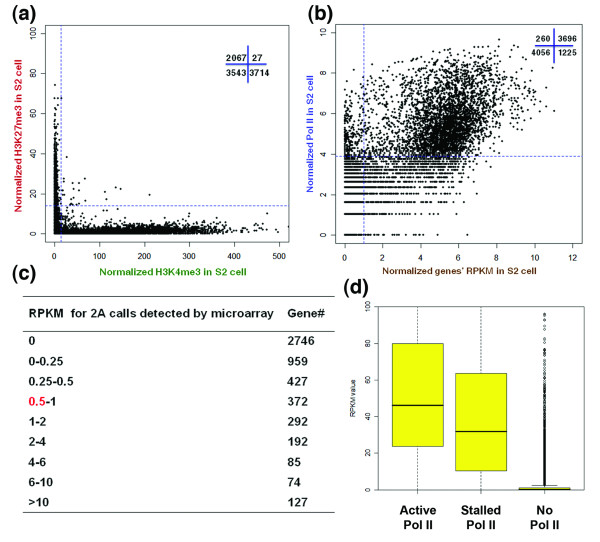
**Comparison of RNA-seq and ChIP-seq data using S2cells**. **(a) **Scatter plot for H3K4me3 and H3K27me3 enrichment of all annotated genes. A common 0 to +500-bp window with respect to the TSS was used to calculate both H3K4me3 and H3K27me3 reads. The blue dashed lines indicate the statistical cutoff line - *P *< 0.05 in a 1-kb window per 1 million total reads. Gene numbers in each quadrant are labeled in black. **(b) **Scatter plot for RNA Pol II enrichment and RPKM values for all annotated genes. All labels are the same as in Figure 2d, except that RPKM = 1 is used as a cutoff for expressed genes. **(c) **The RPKM value for genes that have two absent calls (2A) in the microarray. **(d) **Analysis of the RPKM value of transcripts demonstrates that genes with stalled Pol II are weakly transcribed genes. Genes that show an active Pol II profile are expressed at significantly higher levels. Genes with no Pol II profile are expressed at significantly lower levels. The box represents the 25th and 75th percentiles, with the 50th percentile as a black bar. The whiskers refer to outliers that are at least 1.5× the interquartile range from the box. The y-axis represents the RPKM value.

We analyzed the chromatin status in *Drosophila *S2 cells using antibodies against RNA Pol II, H3K4me3, H3K36me3 and H3K27me3 (Additional file 10; Materials and methods). Sequencing depth analysis indicated that most of these assays almost reached (Additional file 2e,g) or reached (Additional file 2f,h) the detection saturation plateau. For libraries that are not completely saturated, the strongest peaks enriched with the corresponding modified histones or Pol II should be detected first and are therefore identified by our assays. Through analyzing the ChIP-seq data, we identified 3,742 genes with enriched active H3K4me3 and 2,095 genes with enriched repressive H3K27me3 in S2 cells (*P *< 0.05; Materials and methods). Among them, we found only 27 genes enriched in both H3K4me3 and H3K27me3 (Figure 4a; Additional file 7), indicating that bivalency is also not a prevalent chromatin signature for genes in S2 cells. To compare the ChIP-seq data with the gene expression level, we profiled the S2 cell transcriptome using the RNA-seq method (Materials and methods). We found that 25 out of the 27 (93%) bivalent genes in non-synchronized S2 cells are actively expressed (RPKM ≥ 1), indicating that the bivalency may be a result of temporally regulated gene expression during cell cycle. Our S2 cell data are consistent with a previous report using fly embryos [38], where the authors identified 4,893 H3K4me3- and 2,480 H3K27me3-enriched regions, with only 161 overlapping regions. The even fewer bivalent genes in S2 cells (27 genes) than those in *bam *testis (91 genes) may reflect a higher homogeneity of cultured cells than of dissected tissues. We also identified 3,956 genes that have significant binding of RNA Pol II at their promoter region (*P *< 0.05), and 5,281 genes that are unbound by Pol II (Figure 4b). We found that among all the Pol II-enriched genes, 93% are expressed genes (RPKM ≥ 1; Figure 4b). In contrast, only 260 genes that were expressed at very low to undetectable levels (RPKM <1) have enriched Pol II binding at their promoter region; these genes comprise approximately 6.6% of the entire Pol II-enriched genes and 2.7% of the total genes analyzed (9,459 genes). Consistently, more than 99.8% of H3K4me3-enriched genes are actively expressed (3,720 genes out of 3,728 genes with significant enrichment of H3K4me3 have RPKM values ≥ 1; Additional file 11). When we compared the gene expression profiles in S2 cells determined by microarray versus RNA-seq techniques, we found that 770 genes considered as silent based on microarray analysis (two 'absent' calls in both biological replicates) were actually expressed according to their RPKM values (RPKM ≥ 1; Figure 4c) in the RNA-seq data set.

We next applied the ChIP-seq data using antibodies against RNA Pol II to compute the stalling index of individual genes. Through this assay, we identified 1,821 genes with stalled Pol II in S2 cells (Additional file 9a; Materials and methods and [39]). We compared these 1,821 genes with the genes with promoter-proximal enrichment of polymerase, which were identified in the previous study using S2 cell [51]. In fact, approximately 86.2% genes with promoter-proximal enrichment of polymerase overlapped with the genes with stalled Pol II identified in our analysis (Additional file 9a), indicating that the criteria we used to define genes with stalled Pol II is consistent with previous studies. Ontology assays further confirmed that the significant categories (*P *< 0.01) of genes identified using either method are similar (Additional file 9c,d). Noticeably, compared to genes with active Pol II, the genes with stalled Pol II have lower expression levels (Figure 4d), although most (88.5%) of them are actively expressed (RPKM ≥ 1). These results suggest that Pol II binding correlates well with active gene expression status. More sensitive techniques, such as RNA-seq, can accurately detect transcript level for comparison with chromatin structure, in order to understand how epigenetic mechanisms regulate gene expression. To further confirm that our results are not algorithm-specific, we re-analyzed the data with an independent method, the SICER software [52] (Materials and methods). This software uses different algorithms to identify Pol II or modified histone-enriched genomic regions, regardless of their associations with genes. The SICER software has been used to identify bivalent domains in multipotent hematopoietic stem cell lineages in mammals [31,32]. Using SICER, we confirmed that most H3K4me3- and H3K27me3-enriched regions are not overlapping (data not shown) and therefore are monovalent in *Drosophila bam *testis and cultured S2 cells. Using SICER, we also confirmed that most genes associated with Pol II-enriched regions in these two samples are actively expressed (data not shown).

### Does cell type specificity dictate chromatin architecture?

**Figure 5:**
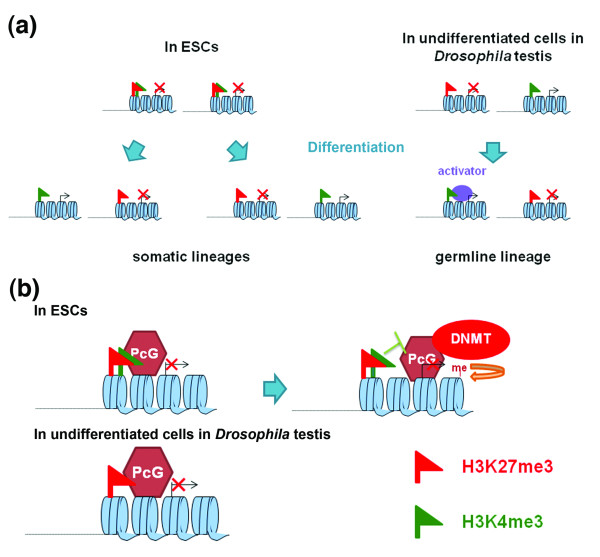
**Cartoons comparing ESCs and undifferentiated cells of *bam *testis**. **(a) **During ESC differentiation, bivalent genes resolve into monovalent genes according to cell type specificity in somatic lineages. In the *Drosophila *male germline lineage, monovalent genes in undifferentiated cells may either retain their chromatin signature or switch to another pattern. Differentiation genes that are required for spermatogenesis require additional activation mechanisms to turn on their expression robustly, in addition to the removal of the repressive H3K27me3 mark. **(b) **A potential molecular mechanism that renders the bivalency and poised status dispensable in the *Drosophila *germline stem cell lineage, due to the lack of endogenous DNA methylase activity. DNMT, DNA methyltransferase.

One possible reason for the different chromatin features identified in our data versus previously published mammalian studies is cell type differences. The *bam *testis we used for ChIP-seq analysis is mainly enriched with transit-amplifying spermatogonial cells, and the chromatin differences we found could come from the distinction between transit-amplifying cells and *bona fide *stem cells. Another possibility for such differences between undifferentiated germ cell-enriched testis samples and ESCs is that germ cells are part of a unilineage stem cell system, but ESCs are pluripotent. Thus, in the germline lineage, differentiation genes have a unidirectional switch from silent to activated status during spermatogenesis, whereas developmental genes in ESC lineages have multi-directional switches during lineage-specific differentiation. The bivalent signature of ESC genes may enable more dynamic and refined activation upon perceiving additional signals (Figure 5a). Indeed, a permanganate footprint assay using early staged *Drosophila *embryos, which are pluripotent, showed evidence of paused Pol II at several genes. In contrast, there was no such evidence when using S2 cells derived from older embryos, at which stage the pluripotency may be lost [39]. Alternatively, these differences may simply reflect distinctive features between somatic cells and germ cells. Germ cells give rise to gametes, which are the most 'immortal' cell type due to their ability to produce the next generation of an entire organism upon fertilization [53]. This requirement to reset totipotency may distinguish germ cells from somatic cells with regard to their chromatin features (Figure 5a).

### Does species specificity dictate chromatin architecture?

The chromatin structure we observed in undifferentiated germ cell-enriched *bam *testis may also apply to *Drosophila *S2 cells, raising another possibility that species difference should be considered. It has been demonstrated that bivalent genes in mammalian ESCs are also enriched with PcG activities [10-12]. These PcG components have the capability to recruit DNA methyltransferases to methylate DNA and silence gene expression in a more permanent manner [54-56]. In contrast, the active H3K4me3 mark placed by the TrxG complex [4-6] may keep chromatin in a relatively open state by recruiting RNA Pol II and initiating low level transcription to prevent DNA methylation (Figure 5b). Indeed, many bivalent domains in ESCs reside at the highly conserved non-coding elements, which are enriched with CpG islands and potentiated for DNA methylation [3]. In contrast to mammalian systems, the DNA methylase activity is almost negligible in adult flies [57-60]. Therefore, differentiation genes in *Drosophila *are not subject to a 'stable' repression caused by DNA methylation as in the mammalian systems, which may promote more dynamic gene regulation while rendering bivalency and paused RNA Pol II status dispensable in *Drosophila*. Noticeably, recent work in *Xenopus *embryos also demonstrated that active H3K4me3 and repressive H3K27me3 regulate distinct groups of genes in a spatially controlled manner. It will be interesting to study monovalency versus bivalency during animal development, as well as evolution.

## Conclusions

Recent data have suggested two non-mutually exclusive mechanisms of gene expression regulation, paused Pol II and bivalent modifications, in mammalian ESCs and other stem or progenitor cells. In these primitive cell types, developmental regulators are characterized by bivalent domains harboring both the H3K4me3 and H3K27me3 marks [3]. These genes may also be bound by paused Pol II, which may poise them for rapid induction during differentiation or in response to developmental and environmental stimuli [2,3,12]. Here our analysis of undifferentiated-cell-enriched *Drosophila bam *testis, by combining highly sensitive ChIP-seq and RNA-seq methods, indicates that most differentiation genes are not enriched with the bivalent signature. In addition, these genes are unpoised with no significant binding of paused Pol II. These data suggest that transcription of differentiation genes in this system is mainly controlled at the Pol II recruitment step but not at the elongation step. These chromatin signatures we identified could reflect cell type specificity, species specificity, or both.

## Materials and methods

### Fly strains and husbandry

Flies were raised using standard medium at 25°C unless stated otherwise. The *bam*^1^/TM3 stock was obtained from Bloomington *Drosophila *Stock Center. The *bam*^*114-97*^/TM6B stock was a gift from Dr Margaret Fuller. The *bam*^*1*^*/bam*^*114-97 *^testis was used for immunostaining with antibodies against a germ cell-specific mark Vasa [61] and a somatic cell-specific mark Traffic jam (Tj) [62] to demonstrate that most of the cells in this tissue are germ cells (Additional file 12).

### Culturing S2 cells

The *Drosophila *S2 cells (ATCC, CRL-1963, Lot#5054622, Manassas, VA, USA) were cultured following the manufacturer's protocol. Briefly, one vial of S2 cell stock was taken from liquid nitrogen and thawed quickly at room temperature. Once the cells were completely thawed, they were transferred to a 25 cm^2 ^flask (Corning, CLS430639-20EA, Lowell, MA, USA) containing 5 ml of room temperature complete Schneider's *Drosophila *medium (GIBCO, #11720-034, Carlsbad, CA, USA; contains 10% heat-inactivated fetal bovine serum (GIBCO #16140-071)). After incubating at 25°C for 30 minutes, the S2 cells were centrifuged at 1,000 rpm, the medium was removed and the cells were transferred into a 25 cm^2 ^flask (Corning, CLS430639-20EA) containing 5 ml of room temperature complete Schneider's *Drosophila *medium. The S2 cells were harvested at the exponentially growing stage after incubating at 25°C for about 90 hours.

### Microarray experiments and data analysis

Total RNA from approximately 200 pairs of *bam *(*bam*^*1*^/*bam*^*114-97*^) fly testes was extracted using TRIzol (Invitrogen, #15596-018, Carlsbad, CA, USA) following the manufacturer's instructions and the genomic DNA was degraded using 2 Units of DNase I (Fermentas, #EN0521, Glen Burnie, MD, USA) at 37°C for 20 minutes. Total RNA from *Drosophila *S2 cells was extracted using the Qiagen RNeasy Mini Kit (catalogue number 74014, Valencia, CA, USA), and the genomic DNA was degraded with RNase-Free DNase (catalogue number 79254). RNA integrity was checked by gel electrophoresis (1% agarose). Approximately 4 μg of total RNA from each biological replicate were used to generate labeling probes to hybridize with the Affymetix GeneChip *Drosophila *Genome 2.0 Array according to the Affymetrix protocol. Three biological replicates were performed for *bam *testes and two biological replicates for S2 cells.

Microarray hybridization was processed at the Genomics Core Facility at the National Heart, Lung and Blood Institute and the raw data were exported from the Affymetrix Microarray Suite (MAS). The CEL files were used for signal normalization with RMA as part of the limma package from the Bioconductor R packages [63]. The 'Present (P)', 'Absent (A)' and 'Marginal (M)' calls were retrieved with the code of 'eset' in the Affy package.

### RNA-seq

Extraction of RNA using *bam *testes or S2 cells was performed using similar methods as described for microarray experiments. For the total RNA from *bam *testes (8.5 μg) and S2 cells (approximately 20 μg), we performed two rounds of mRNA isolation using Dynabeads mRNA purification kit (Invitrogen, #610-06), according to the manufacturer's instructions. The final mRNAs were eluted in 13.5 μl 10 mM Tris-HCl (pH 7.5) and immediately used to generate the first strand cDNA, using 4 μl random hexamers (ABI, #N8080127, Foster City, CA, USA) and SuperScript II Reverse Transcription Kit (Invitrogen, #18064-014) in a 30 μl final volume, following the manufacturer's instructions. The second strand cDNA was generated with the following recipe: 10 μl 5× second strand buffer (500 mM Tris-HCl pH7.8, 50 mM MgCl_2_, 10 mM DTT), 30 nmol dNTPs (Invitrogen, #18427-013), 2 Units of RNase H (Invitrogen, #18021-014) and 50 Units of DNA Pol I (Invitrogen, #18010-025). The entire reaction mix was incubated at 16°C for 2.5 hours. The double-stranded DNA (dsDNA) was purified with a QIAquick PCR purification kit (Qiagen, #28106) and the concentration was quantified using a Qubit fluorometer (Invitrogen).

To generate sequencing libraries, about 300 ng dsDNA from each sample was fragmented by sonication using Bioruptor (Diagenode, UCD-200-TM-EX, Sparta, NJ, USA) using medium power output for 30 minutes in ice water. The resulting DNA fragments were analyzed by agarose gel to verify they were within the approximately 100 to 300 bp size range. Sequencing libraries were prepared as follows: end-repair (DNA end-repair kit from Epicenter, #ER0720, Madison, WI, USA); A-tailing (300 ng dsDNA, 5 μl Thermo buffer, 10 nmol dATP, 15 Units of Taq polymerase, at 70°C for 30 minutes); Solexa adaptor ligation (300 ng dsDNA, 4 μl DNA Ligase buffer, 1 μl Solexa adaptor mix, 3 ul DNA Ligase, at 70°C overnight); PCR (98°C 10 s, 65°C 30 s, 72°C 30 s for 16 cycles; then additional 72°C for 5 minutes) amplification with adaptor primers and size selection (200 to 400 bp). Then the library dsDNA for S2 cells was used on an Illumina Genome Analyzer II at a concentration of 10 ng per lane.

We obtained 20,041,035 and 9,780,523 total reads from an Illumina Genome Analyzer II for *bam *testis and S2 cell samples, respectively. And 10,163,916 (*bam *testis) and 6,263,318 (S2 cell) unique and non-redundant reads were used for downstream data analysis.

The Gene Expression Omnibus accession number for the raw and analyzed RNA-seq data is [GEO:GSE19325].

### Comparison of microarray results with RNA-seq data

To compare the RPKM value from RNA-seq data with microarray results, we first retrieved 12,728 *Drosophila *genes from the Ensembl database, which also have probe(s) in the Affymetix GeneChip *Drosophila *Genome 2.0 Array. Genes with multiple probes were filtered out if different probes gave inconsistent 'Present (P)' or 'Absent (A)' calls. We then analyzed the RPKM distribution for genes with all 'Absent' calls or all 'Present' calls in microarray datasets, for both *bam *testis and S2 cell samples.

The histograms were generated using the 'hist' function in the R programming environment (R version 2.5.0 [64]). To calculate the log_2 _RPKM values of individual genes, all their original RPKM values were added a pseudo-count of 1.

### Chromatin immunoprecipitation

For each modified histone and Pol II ChIP experiment, we dissected approximately 200 pairs of *bam *testes in cold phosphate-buffered saline (PBS) and grouped them in 200 μl PBS that contained protease inhibitor (Roche complete mini, #11836153001, Nutley, NJ, USA) and 0.5 mM phenylmethanesulfonyl fluoride (PMSF; MP Biomedicals, #195381, Solon, OH, USA). Approximately 1,000 cells could be extracted from one *bam *testis. We then added 5.5 μl 37% fresh formaldehyde (Supelco, #47083-U, Bellefonte, PA, USA) and incubated at 37°C for 15 minutes. The testes were washed twice with 450 μl cold 1× PBS (with inhibitors and PMSF). Then 200 μl lysis buffer (50 mM Tris-HCl, pH7.6, 1 mM CaCl_2_, 0.2% Triton X-100, 5 mM butyrate, 1× protease inhibitor cocktail, and 0.5 mM fresh PMSF) was added and the tissues were homogenized thoroughly followed by incubation at room temperature for 10 minutes. Chromatin was sheared into approximately 200-bp fragments by sonication using Microtip (Misonix, Inc., Microson XL-2000, Farmingdale, NY, USA) with the following procedure: 4 s at power 20, rest for 50 s, 4 to 5 times, followed by spinning at 14k rpm for 10 minutes at 4°C. The chromatin was diluted 10× with RIPA buffer (10 mM Tris, pH7.6, 1 mM EDTA, 0.1% SDS, 0.1% Na-Deoxycholate, 1% Triton X-100, with protease inhibitors and PMSF) and 50 μl of this dilution was reverse cross-linked with 0.25 M NaCl for 2 hours at 65°C and used as input for real-time PCR analysis.

We washed 40 μl of Dynabeads Protein A (Invitrogen, #100.01D) with 600 μl 1× PBS. We then added 100 μl 1× PBS with 4 μg antibody and incubated the antibody-Protein A beads mixture at room temperature for 40 minutes with occasional tapping. After the unbound antibody was removed using the manipulator (Invitrogen, DYNAL MPC-S), 1 ml of the chromatin extract was added to the beads and the mixture was rotated at 4°C overnight. The beads were then washed twice with 1 ml RIPA buffer, twice with 1 ml RIPA buffer containing 0.3 M NaCl, once with LiCl wash buffer (0.25 M LiCl, 0.5% NP40, and 0.5% sodium deoxycholate), once with 1 ml TE (10 mM Tris-HCl, pH 8.0 and 1 mM EDTA) containing 0.2% Triton X-100, and once with 1 ml TE. The beads were then suspended in 100 μl 1× TE containing 3 μl 10% SDS and 5 μl 20 mg/ml proteinase K, followed by incubation at 65°C overnight. After the supernatant was collected, the beads were washed once more with 100 μl TE with 0.5 M NaCl. The supernatant from this wash was combined with the previous supernatant. The combined samples were treated by Phenol/Chloroform extraction, salt/EtOH precipitation, and dissolved in 50 μl 1× TE. The products were either used for real-time PCR analyses or processed for Solexa sequencing according to the established protocol. Antibodies used include those against H3K4me3 (Abcam, #ab8580, Cambridge, MA, USA), H3K27me3 (Millipore, #07-449, Billerica, MA, USA), H3K36me3 (Abcam, #ab9050), H3 (Abcam, #ab1791) and RNA Pol II (Abcam, ab5408).

### ChIP experiment using S2 cells

Exponentially growing S2 cells were harvested and dissolved in digestion buffer (50 mM Tris-HCl, pH7.6, 1 mM CCl_2_, 0.2% Triton X-100, 5 mM butyrate, 1× protease inhibitor cocktail and 0.5 mM PMSF). Chromatin was prepared and ChIP-seq experiments were performed as described previously [27] with antibodies against Pol II (Abcam, #ab5408), H3K4me3 (Abcam, #ab8580), H3K27me3 (Millipore/Upstate, #07-449), and H3K36me3 (Abcam, #ab9050).

### Solexa pipeline analysis

The 25-bp sequencing reads were obtained from the Illumina Genome Analyzer pipeline. All reads were aligned to the *Drosophila *genome (dm3) using the ELAND (Efficient Local Alignment of Nucleotide Data) software, allowing up to two mismatches with the reference sequence. Only uniquely mapped reads were retained. For multiple identical reads, at most three copies were retained to reduce the possibility of biases from PCR amplification. The output of the Genome Analyzer pipeline was converted to browser extensible data (BED) files. The wig files used for visualization on the UCSC browser were generated from the uniquely mapped reads using a 4-bp window and 160 bp as the DNA fragment size, as previously described [27]. The size of the DNA fragment was determined by the distance from the 5' to the 3' peak of the mapped reads, as shown in Additional file 13.

The Gene Expression Omnibus accession number for the raw and analyzed ChIP-seq data is [GEO:GSE19325].

### Defining genomic regions for analyzing modified histone and Pol II enrichment

To determine the regions used for modified histone and Pol II occupancy of the annotated transcripts in Figure 1, we first plotted each histone modification and Pol II using the entire annotated transcripts that are applicable for ChIP-seq analysis. Based on the overall enrichment plot, we used the region from -250 to +250 bp (the TSS was defined as 0) to calculate the Pol II enrichment of individual transcripts. A 0 to +500-bp window was used for H3K4me3 and H3K27me3, and a +500 to 1,500-bp window was used for H3K36me3 enrichment calculations. For genes with a transcript size longer than 1 kb, a +500 to +1,000-bp window was defined as the gene body region to calculate the stalling index.

### *Drosophila *genes used for ChIP-seq analysis

*Drosophila *genes used for ChIP-seq analysis were derived from the UCSC database (April 2006/BDGP R5/dm3), which contained 14,058 genes and 21,243 transcripts. The coordinates for these transcripts were downloaded (August 2009) from the UCSC table browser [65]. Compared to human and mouse genomes, the *Drosophila *genome is more gene-dense, including many overlapping genes and short distances between TSSs, which may lead to incorrect conclusions from ChIP-seq analysis. To avoid this, we chose transcripts for analysis using the following three steps. First, exclude transcripts shorter than 500 bp (transcript size is defined as the distance between the annotated transcription start and end sites) because these short transcripts will affect the promoter analysis for H3K4me3 and H3K27me3. A total of 740 transcripts (736 genes) were excluded using this criterion. Second, exclude overlapping transcripts from different genes (Additional file 14) and transcripts that have short distances between their TSSs; 6,497 transcripts (4,535 genes) overlap with at least one other transcript, but 3,832 transcripts (2,555 genes) among them were useful for promoter analysis (-250 to +250 bp, or 0 to +500 bp), because the overlapping regions do not affect promoter analysis of these genes. A total of 3,558 non-overlapping transcripts (2,729 genes with opposite transcription direction) have a distance between TSSs shorter than 400 bp, which will affect the Pol II promoter analysis (-250 to +250 bp), and were thus excluded. Third, after removal of certain transcripts using the above two criteria, 14,849 transcripts (9,459 genes) were retained for ChIP-seq promoter analysis. In total, 7,799 genes were applicable for stalling index analysis, and 6,400 genes for H3K36me3 enrichment analysis. Among these 9,459 genes, 2,612 have multiple transcripts, but only one transcript for each was used for ChIP-seq analysis.

### Choosing the dominant transcript for multi-isoform genes

For genes with multiple isoforms, we used the most abundant one for the comparison between ChIP-seq and RNA-seq data.

#### *bam *Pol II versus testis RPKM, and S2 Pol II versus cell RPKM

1, The dominant transcript was determined as that with the highest number of Pol II ChIP-seq reads in the -250 to +250-bp window with respect to the TSS region. If multiple transcripts have the same number of reads in this region, criterion 2 is used. 2, For transcripts that are longer than 1 kb, the dominant transcript was determined as that with the highest number of ChIP-seq reads in the gene body (+500 to +1,000-bp with respect to the TSS). For those transcripts that are shorter than 1 kb, the dominant transcript was determined as that with the highest number of ChIP-seq reads for H3K4me3 in the region 0 to +500 bp with respect to the TSS. If multiple transcripts have the same numbers of reads, criterion 3 is used. 3, The dominant transcript was determined as that with the longest transcript. If all isoforms have the same length, one was chosen randomly.

#### *bam *H3K4me3 versus H3K27me3, bam H3K4me3 versus testis RPKM, S2 cell H3K4me3 versus H3K27me3, and S2 H3K4me3 versus RPKM

1, The dominant transcript was determined as that with the highest number of H3K4me3 ChIP-seq reads in the region 0 to +500 bp with respect to the TSS. If multiple transcripts have the same number of reads, criterion 2 is used. 2, For transcripts that are longer than 1.5 kb, the dominant transcript was determined as that with the highest number of ChIP-seq reads for H3K36me3. For transcripts that are shorter than 1.5 kb, and thus are not applicable to calculate H3K36me3 reads, the dominant transcript was determined as that with the highest number of ChIP-seq reads for Pol II in the region -250 to +250 bp with respect to the TSS. If multiple transcripts have the same number of reads, criterion 3 is used. 3, The dominant transcript was determined as that with the longest transcript. If all isoforms have the same length, one was chosen randomly.

#### *bam *H3K36me3 versus testis RPKM, and S2 cell H3K36me3 versus RPKM

1, For the 6,400 genes that have at least one transcript longer than 1.5 kb, the dominant transcript was determined as that with the highest number of H3K36me3 ChIP-seq reads in the region +500 to +1,500 bp with respect to the TSS. If multiple transcripts have the same number of reads, criterion 2 is used. 2, The dominant transcript was determined as that with the highest number of ChIP-seq reads for H3K4me3 in the region 0 to +500 bp with respect to the TSS. If multiple transcripts have the same number of reads, criterion 3 is used. 3, The dominant transcript was determined as that with the longest transcript. If all isoforms have the same length, one was chosen randomly.

### Comparison of ChIP-seq results with the RNA-seq data

To generate the plots in Figure 1, we retrieved annotated *Drosophila *genes from the Ensembl database for ChIP-seq analysis. We classified them into silent and expressed genes according to RPKM value (genes with RPKM = 0 were classified as the silent group, and genes with RPKM ≥ 1 were classified as the expressed group). The expressed group was further classified into low, moderate and high groups based on RPKM values. The coordinates of these transcripts were downloaded from the UCSC table browser [65]. The read density was calculated in a 5-bp window across the genome.

### Comparison of our ChIP-seq data with the published ChIP-chip data

We compared our 91 bivalent genes in *bam *testis with the ChIP-chip data using BG3 and D23 cells [37]. We first downloaded both the H3K4me3 and H3K27me3 sgr files from the NCBI website. We retained the probes whose ChIP/input hybridization intensity ratios are ≥ 2, to be consistent with the authors' definition of enriched regions. We then mapped the probes to the promoter regions (0 to +500 bp) of our 91 bivalent genes. For BG3 cells, we found 6 out of the 91 *bam *testis bivalent genes contained an enriched H3K4me3 signal; and 4 of these 91 bivalent genes contained an enriched H3K27me3 signal. However, none of these 91 bivalent genes is enriched with both H3K4me3 and H3K27me3 in BG3 cells. Similarly, for D23 cells, 5 and 11 out of these 91 *bam *testis bivalent genes contained enriched H3K4me3 and H3K27me3 signals, respectively. Again, none of these 91 bivalent genes is enriched with both H3K4me3 and H3K27me3 in D23 cells.

We also checked the ChIP-on-chip data using *Drosophila *embryos [38] and found 4,893 H3K4me3 and 2,480 H3K27me3 enriched regions. However, only 161 of them overlap with each other. We then compared the 91 bivalent genes we identified in *bam *testis with these 161 bivalent regions in embryos. From this comparison, we found only two genes (*CG4637 *and *CG9610*) for which the promoter region (0 to +500 bp) overlaps two bivalent regions in embryos (chr3R: 18965718-18967662 and chr3R: 4158335-4159501).

### Determination of the *P*-value of enrichment of ChIP-seq reads within a 500-bp window

Since we used a 500-bp window to detect enrichment of Pol II and histone modifications (for example, active H3K4me3 and repressive H3K27me3), a sequencing read count threshold was chosen according to the Poisson distribution, which distributes the total and unique reads randomly across the *Drosophila *genome that can be mapped. For every 500-bp window with read number ranging from 1 to 99, the *P*-value was calculated. The threshold was chosen as the minimal number of reads that reached a significant enrichment compared to the random distribution (that is, *P *< 0.05). For example, for the total 1,342,075 reads of anti-Pol II ChIP-seq in *bam *testis, a 500-bp window containing 11 read counts has a *P*-value of 0.03, which passes the threshold *P*-value of ≤ 0.05. We then set the threshold of significant enrichment within a 500-bp window in this data set to be 11 read counts.

### Analysis of the RNA Pol II stalling index

To analyze the RNA Pol II stalling index, we modified a method that was adapted from published work [39,66]. Basically, the stalling index reflects differential Pol II binding at the promoter region versus the gene body region. Therefore, we defined the -250 to +250-bp region around the TSS as the promoter region (with respect to the TSS), and the +500 to +1,000-bp region (with respect to the TSS) as the gene body region. To calculate the stalling index, we first counted the total Pol II reads at the promoter region and at the gene body region. A stalling index was defined as the ratio of total reads in the promoter region divided by the total reads in the gene body region. Based on the stalling index, we classified genes into active, stalled or no Pol II categories based on the following criteria: active Pol II genes, stalling index ≤ 3 and significant Pol II enrichment (*P *< 0.05); stalled Pol II genes, stalling index ≥ 5 and significant Pol II enrichment (*P *< 0.05); no Pol II genes, no significant Pol II enrichment (*P *> 0.05).

### Scatter plot analysis

The scatter plots delineate comparisons of different chromatin modifications in *bam*. All plots were generated in the R programming environment (R version 2.5.0 [64]). Transformation to the log value was used to compare chromatin modifications with RPKM values.

### Box plot analysis

The distribution of gene expression level was analyzed using box plots in the R programming environment (R version 2.5.0 [64]). The box represents the 25th and 75th percentiles, with the 50th percentile as a black bar. The whiskers refer to outliers that are at least 1.5× the interquartile range from the box. The y-axis represents the RPKM value.

### Identification of Pol II or modified histone-enriched regions using SICER

To identify the significantly enriched regions, we used SICER software [52] with the following parameters: window size = 200 bp, gap size = 0 bp and E-value = 100 for Pol II and H3K4me3; window size = 400 bp, gap size = 0 bp and E-value = 100 for H3K27me3 and H3K36me3. The reason we do not allow any gap is due to the density of genes in the *Drosophila *genome.

### Sequencing depth analysis

To analyze the sequencing depth of ChIP-seq, we first shuffled the reads and their corresponding genomic loci, then extracted subsamples (2.5%, 5%, 7.5%, and so on until 100% of the total unique reads), and then identified the enriched regions in each subsample using SICER software as described previously. The E-value was increased from the first subsample (E-value = 3) to the last subsample (E-value = 120) by an increment of 3. We then plotted the correlation between subsamples and the enriched Pol II or modified histones in the corresponding regions, as shown in Additional file 2.

### Gene Ontology assay

The gene function ontology analyses were performed using the DAVID 2008 informatics tools [67], based on the Gene Ontology Consortium [68]. All Ensembl annotated genes were used as a background comparison. Two particular Gene Ontology annotations (molecular function and biological process) were analyzed with a cutoff P value of < 0.01.

## Abbreviations

*bam*: *bag-of-marbles*; *bgcn*: *benign gonial cell neoplasm*; ChIP: chromatin immunoprecipitation; ChIP-seq: chromatin immunoprecipitation followed by high-throughput sequencing; dsDNA: double-stranded DNA; ESC: embryonic stem cell; *E(z)*: *Enhancer of Zeste*; GSC: germline stem cell; PBS: phosphate-buffered saline; PcG: Polycomb group; PMSF: phenylmethanesulfonyl fluoride; Pol II: RNA Polymerase II; RPKM: sequencing reads per kilobase of exon per million mapped reads; *sa*: *spermatocyte arrest*; TrxG: Trithorax group; TSS: transcriptional start site.

## Authors' contributions

QG, SE, GW, KC and XC performed the experiments; QG and DS analyzed the data; QG, DS, KZ and XC wrote the paper.

## Supplementary Material

Additional file 1 Summary of unique and non-redundant reads in each sample. Click here for file

Additional file 2 **Sequencing depth of ChIP-seq using various antibodies in both *bam *testis and S2 cell samples**. Sequencing depth is analyzed by calculating the enriched regions for each subsample of the corresponding ChIP-seq experiment. The blue line represents the trend line. The x-axis of the plot indicates the percentage of subsample reads compared to the total unique reads, whereas the y-axis indicates the identified islands. Click here for file

Additional file 3 **More examples of the monovalent genes in *bam *testis**. **(a) **Transcription levels of two representative H3K4me3-monovalent genes, *Decondensation factor 31 *(*Df31*) and *Rpd3*, and two representative H3K27me3-monovalent genes, *don juan *(*dj*) and *fuzzy onion *(*fzo*), in *bam *and wild-typetestis, respectively. **(b-e) **UCSC genome browser screenshot showing the active H3K4me3 monovalency at (b) *Df31 *and (c) *Rpd3 *genes, as well as the repressive H3K27me3 monovalency at (d) *dj *and (e) *fzo *genes, respectively. These examples are chosen based on their expression profiles in [21]. Click here for file

Additional file 4 **Validation of bivalent genes using ChIP followed by real-time PCR analyses on five gene loci**. **(a) ***Rpl7A *gene is used as a positive control for the active H3K4me3 enrichment and *don juan *(*dj*) is used as a positive control for the repressive H3K27me3 mark. All five bivalent genes have H3K4me3 and H3K27me3 at levels between the two controls. The y-axis denotes the ChIP-ed DNA/input DNA percentage. Primer pairs used for PCR validation are shown as black boxes in each panel of (b-f). Primer sequence information is in Materials and methods. **(b-f) **UCSC genome browser screenshots showing H3K4me3 and H3K27me3 enrichment at each of the following genes' loci: (b) *bgcn*; (c) *Neurochondrin*; (d) *CG14834*; (e) *NPC2*; and (f) *retn*. Click here for file

Additional file 5 **Bivalent genes in undifferentiated cells in *bam *testis**. In total, 91 bivalent genes were identified in undifferentiated cells in *bam *testis, including 18 differentiation genes (RPKM in *bam *< 0.5, in wild-type ≥ 1), which are highlighted in red. Click here for file

Additional file 6 **Determination of the cutoff RPKM value for expressed genes by comparing microarray and RNA-seq data**. The histogram shows the distribution of RPKMs for genes that are unambiguously called 'Present' in all three replicates from the gene expression microarray data. The RPKM values are adjusted by adding a pseudo-count of 1 prior to the logarithm. Threshold RPKM values of 0.5 and 1 are shown as dashed lines in blue and green, respectively. Approximately 99% of genes that are considered unambiguously 'Present' (3P calls) in the microarray data have an RPKM ≥ 1. Therefore, we assigned RPKM ≥ 1 as the cutoff for expressed genes. Conversely, only 0.4% of these genes have an RPKM < 0.5, and we assigned RPKM < 0.5 as the cutoff for absent or silent genes. Click here for file

Additional file 7 **Bivalent genes in *Drosophila *S2 cells**. In total, 27 bivalent genes were identified in *Drosophila *S2 cells. Click here for file

Additional file 8 **Most genes enriched with H3K4me3 are actively expressed in *bam *testis**. Scatter plot for H3K4me3 enrichment and RPKM values for all annotated genes. All labels are the same as in Figure 2d, except RPKM = 1 is used as a cutoff for expressed genes. Click here for file

Additional file 9 **Comparison of our ChIP-seq data with published ChIP-chip data. (a) **Flow chart to compare our ChIP-seq data in *bam *testis and S2 cells with published ChIP-chip data in S2 cells [51] and embryos [39]. **(b-d) **Ontology analysis of (b) the 695 genes with stalled Pol II in *bam *testis, (c) the 1,821 genes with stalled Pol II in S2 cells, and (d) the 1,014 genes with promoter-proximal enrichment of Pol II [51]. Click here for file

Additional file 10 **Summary of the ChIP-seq results using *Drosophila *S2 cells**. **(a) **The four groups of genes were classified according to their RPKM value based on the RNA-seq results. The numbers in brackets denote genes used for H3K36me3 (K36) analysis. *See Materials and methods for gene selection criteria. **(b-e) **Antibodies used for ChIP-seq are: (b) anti-RNA Pol II (Pol II); (c) anti-H3K4me3 (K4); (d) anti-H3K36me3 (K36); and (e) anti-H3K27me3 (K27). Enrichment of each histone modification and RNA Pol II is plotted over a -5 to +5-kb region with respect to the TSSs of the genes. Click here for file

Additional file 11 **Most genes enriched with H3K4me3 are actively expressed in S2 cells**. Scatter plot of H3K4me3 enrichment and RPKM values for all annotated genes. All labels are the same as in Figure 2d, except RPKM = 1 was used as a cutoff for expressed genes. Click here for file

Additional file 12 Immunostaining of the *bam *testis with antibodies against the germ cell marker Vasa and the somatic marker Traffic jam (Tj). Click here for file

Additional file 13 **Determination of the fragment size for ChIP-seq analysis, based on the distance between the 5' and 3' sequencing read peaks**. All expressed genes (RPKM ≥ 1) were used for the modification level plots over a -2 to +2-kb window with respect to the TSS. **(a-e) **Antibodies used for ChIP-seq in *bam *testis were: (a) anti-RNA Pol II; (b) anti-H3K4me3; (c) anti-H3K36me3; (d) anti-H3K27me3; and (e) anti-H3. Click here for file

Additional file 14 **Cartoons showing overlapping transcripts from different genes that were retained for data analysis**. The transcripts labeled by blue letters were retained for data analysis, in each situation shown for overlapping genes. Click here for file
