# Supplementary figures and images for: Monovalent and unpoised status of most genes in undifferentiated cell-enriched Drosophila testis
Source: Genome Biol. 2010 Apr 15;11(4):R42. doi: 10.1186/gb-2010-11-4-r42 (PMC2884545; doi:10.1186/gb-2010-11-4-r42)

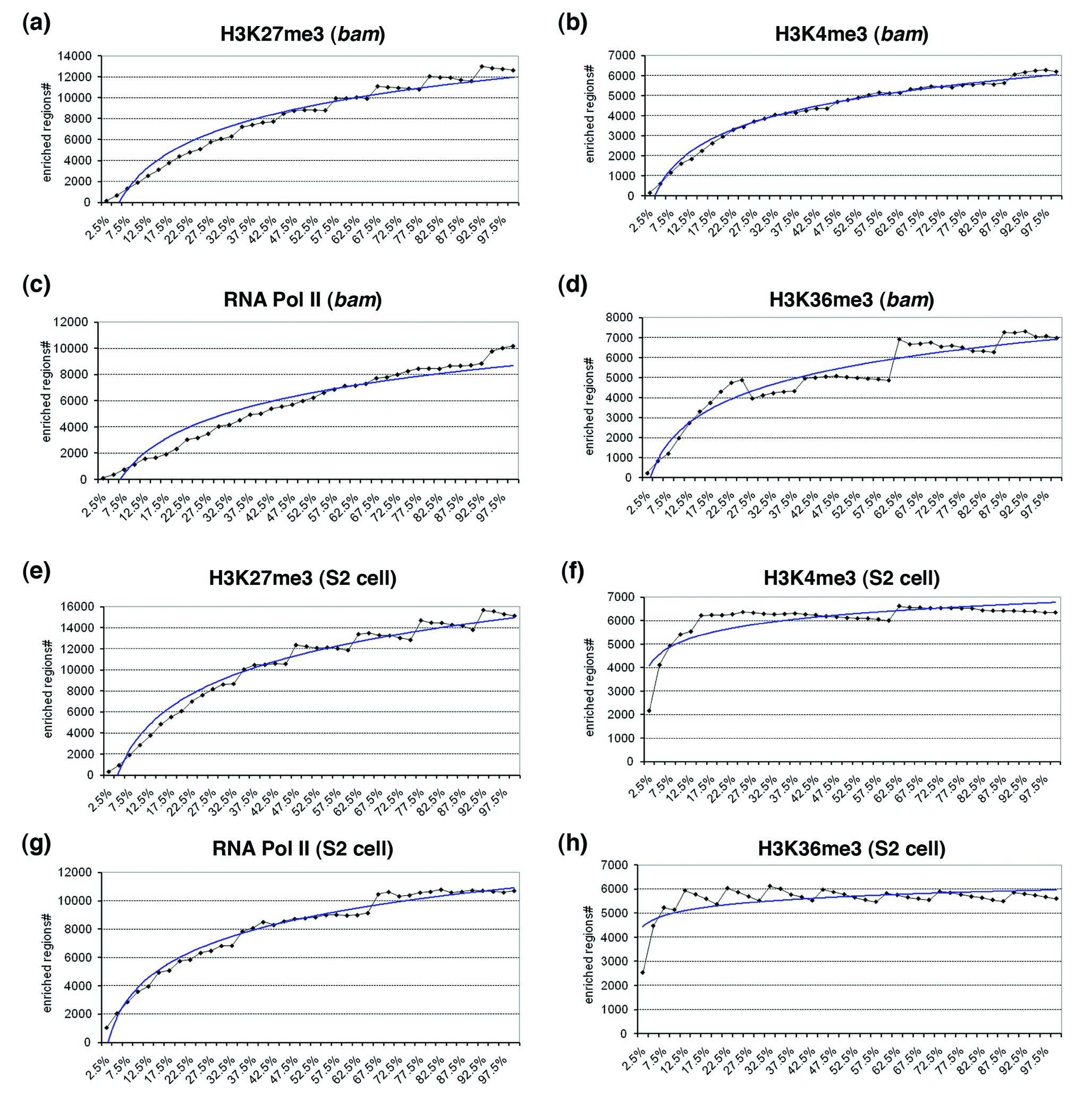

Supplement: Additional file 2 — Sequencing depth of ChIP-seq using various antibodies in both bam testis and S2 cell samples. Sequencing depth is analyzed by calculating the enriched regions for each subsample of the corresponding ChIP-seq experiment. The blue line represents the trend line. The x-axis of the plot indicates the percentage of subsample reads compared to the total unique reads, whereas the y-axis indicates the identified islands. [file gb-2010-11-4-r42-S2.tiff]

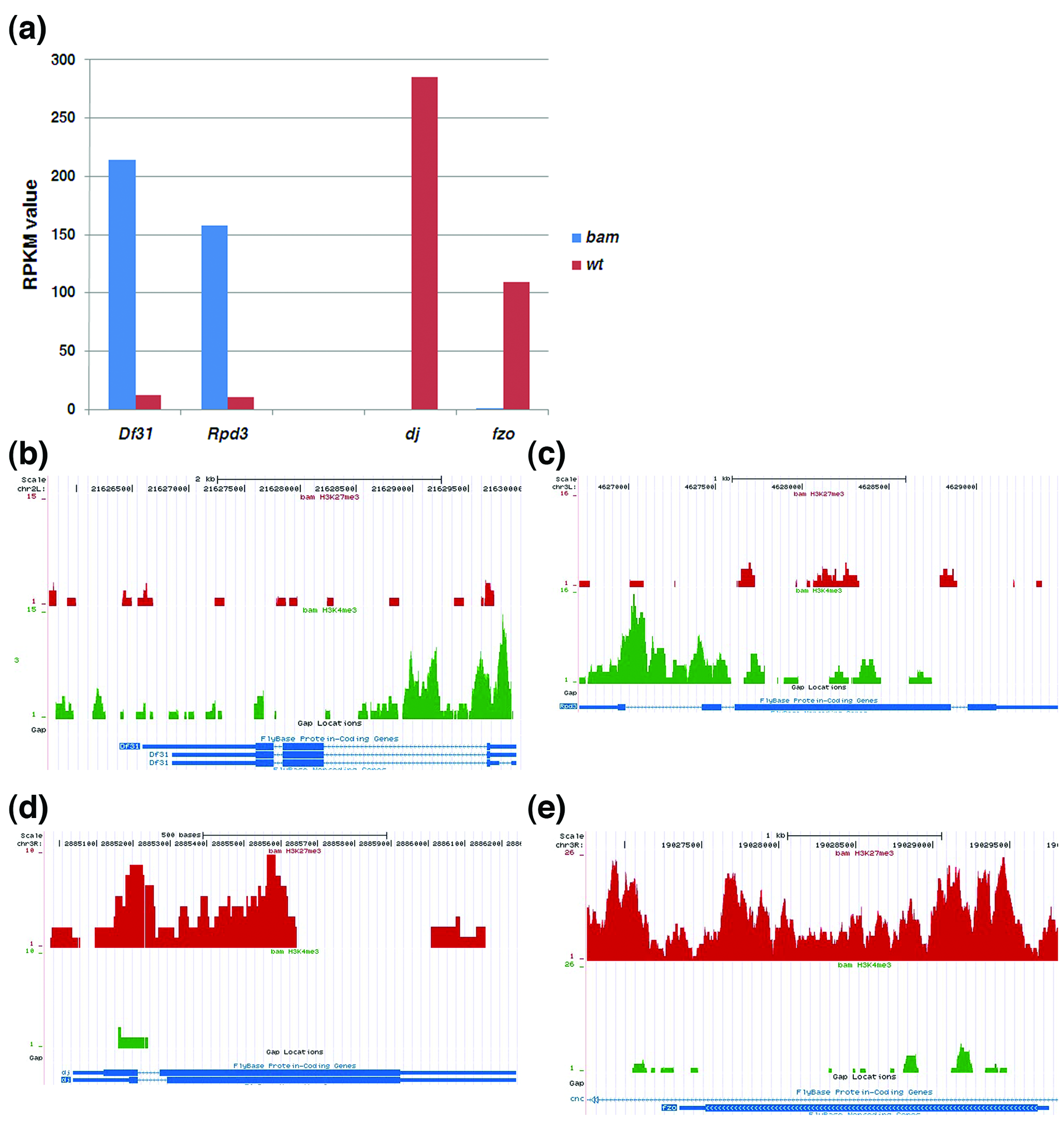

Supplement: Additional file 3 — More examples of the monovalent genes in bam testis. (a) Transcription levels of two representative H3K4me3-monovalent genes, Decondensation factor 31 (Df31) and Rpd3, and two representative H3K27me3-monovalent genes, don juan (dj) and fuzzy onion (fzo), in bam and wild-typetestis, respectively. (b-e) UCSC genome browser screenshot showing the active H3K4me3 monovalency at (b) Df31 and (c) Rpd3 genes, as well as the repressive H3K27me3 monovalency at (d) dj and (e) fzo genes, respectively. These examples are chosen based on their expression profiles in [21]. [file gb-2010-11-4-r42-S3.tiff]

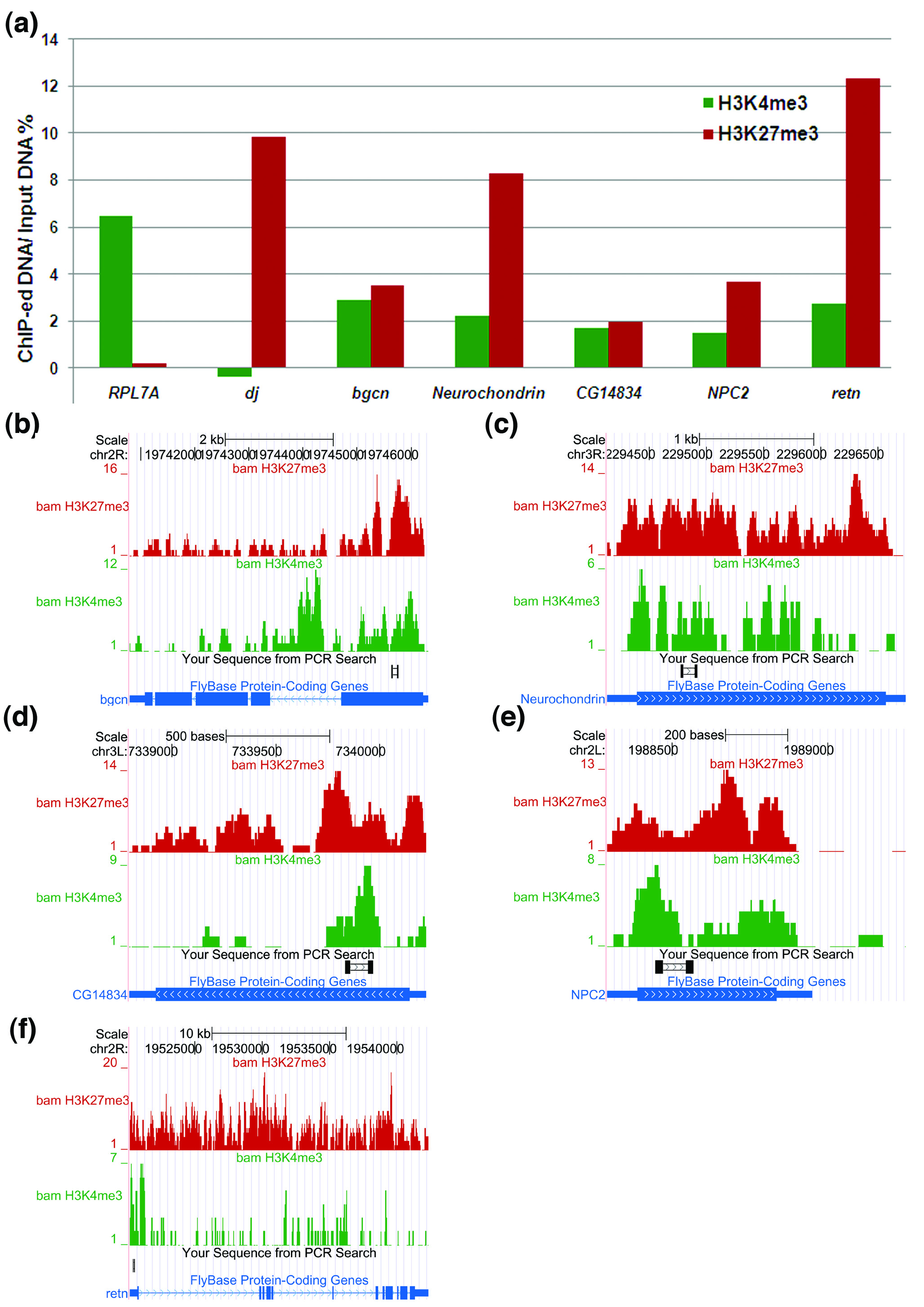

Supplement: Additional file 4 — Validation of bivalent genes using ChIP followed by real-time PCR analyses on five gene loci. (a) Rpl7A gene is used as a positive control for the active H3K4me3 enrichment and don juan (dj) is used as a positive control for the repressive H3K27me3 mark. All five bivalent genes have H3K4me3 and H3K27me3 at levels between the two controls. The y-axis denotes the ChIP-ed DNA/input DNA percentage. Primer pairs used for PCR validation are shown as black boxes in each panel of (b-f). Primer sequence information is in Materials and methods. (b-f) UCSC genome browser screenshots showing H3K4me3 and H3K27me3 enrichment at each of the following genes' loci: (b) bgcn; (c) Neurochondrin; (d) CG14834; (e) NPC2; and (f) retn. [file gb-2010-11-4-r42-S4.jpeg]

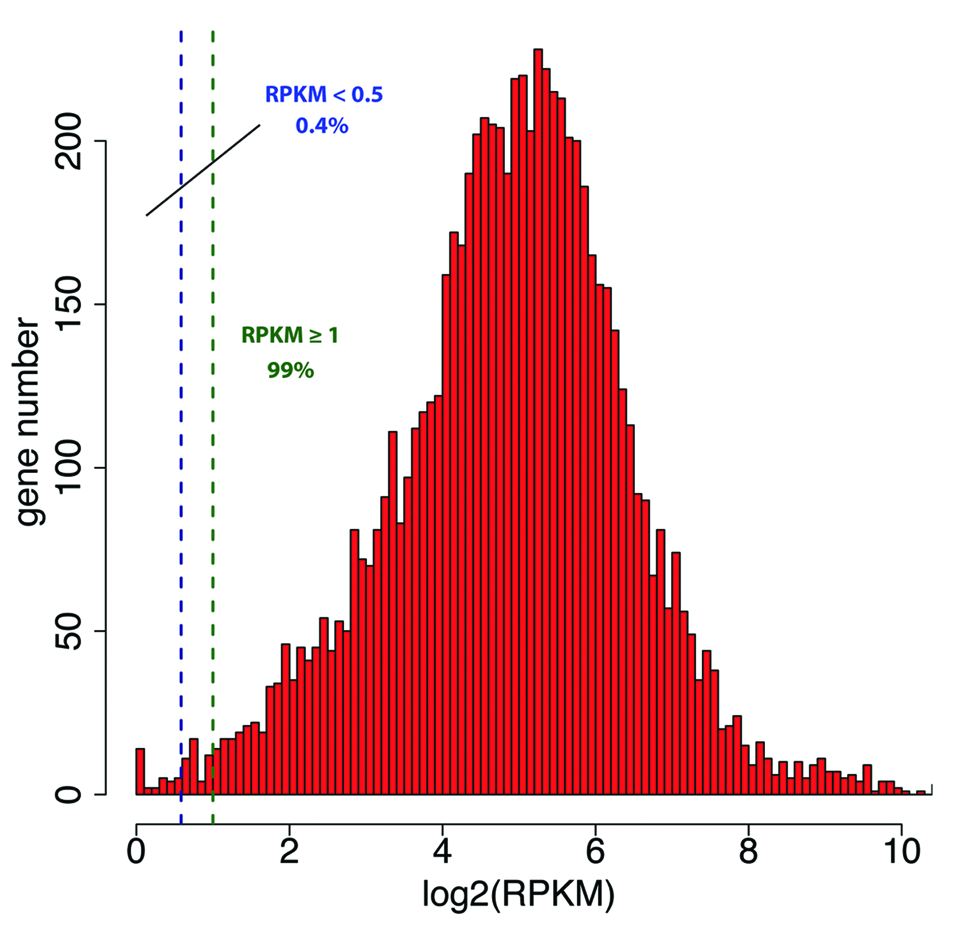

Supplement: Additional file 6 — Determination of the cutoff RPKM value for expressed genes by comparing microarray and RNA-seq data. The histogram shows the distribution of RPKMs for genes that are unambiguously called 'Present' in all three replicates from the gene expression microarray data. The RPKM values are adjusted by adding a pseudo-count of 1 prior to the logarithm. Threshold RPKM values of 0.5 and 1 are shown as dashed lines in blue and green, respectively. Approximately 99% of genes that are considered unambiguously 'Present' (3P calls) in the microarray data have an RPKM ≥ 1. Therefore, we assigned RPKM ≥ 1 as the cutoff for expressed genes. Conversely, only 0.4% of these genes have an RPKM < 0.5, and we assigned RPKM < 0.5 as the cutoff for absent or silent genes. [file gb-2010-11-4-r42-S6.tiff]

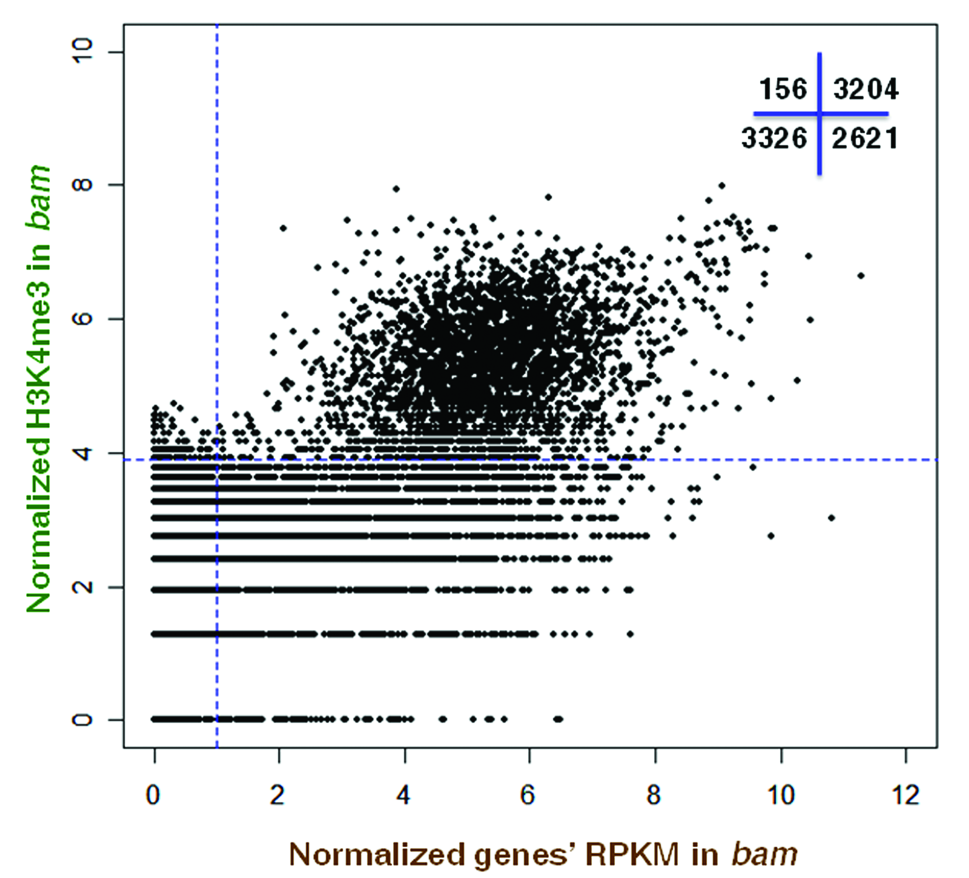

Supplement: Additional file 8 — Most genes enriched with H3K4me3 are actively expressed in bam testis. Scatter plot for H3K4me3 enrichment and RPKM values for all annotated genes. All labels are the same as in Figure 2d, except RPKM = 1 is used as a cutoff for expressed genes. [file gb-2010-11-4-r42-S8.tiff]

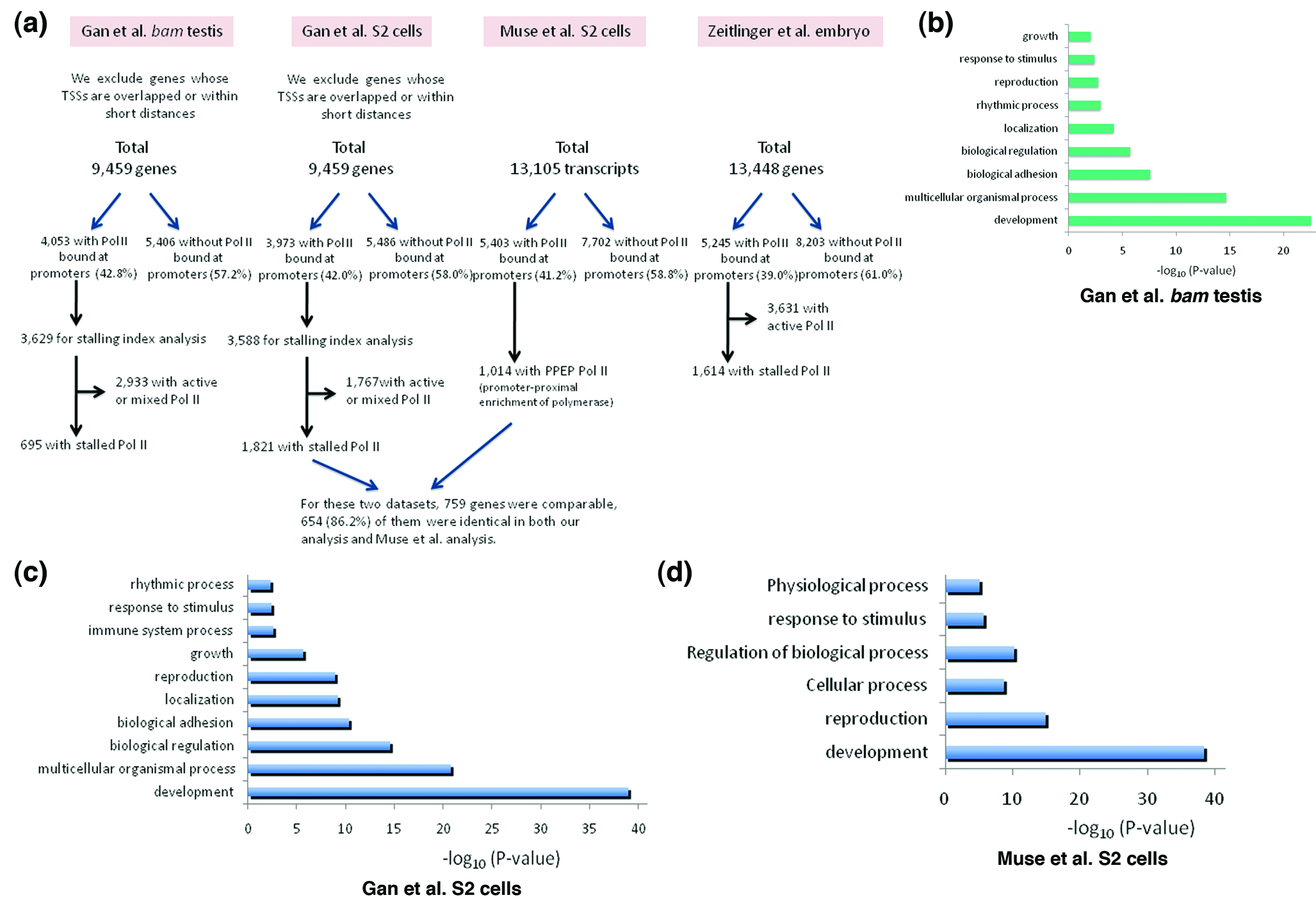

Supplement: Additional file 9 — Comparison of our ChIP-seq data with published ChIP-chip data. (a) Flow chart to compare our ChIP-seq data in bam testis and S2 cells with published ChIP-chip data in S2 cells [51] and embryos [39]. (b-d) Ontology analysis of (b) the 695 genes with stalled Pol II in bam testis, (c) the 1,821 genes with stalled Pol II in S2 cells, and (d) the 1,014 genes with promoter-proximal enrichment of Pol II [51]. [file gb-2010-11-4-r42-S9.tiff]

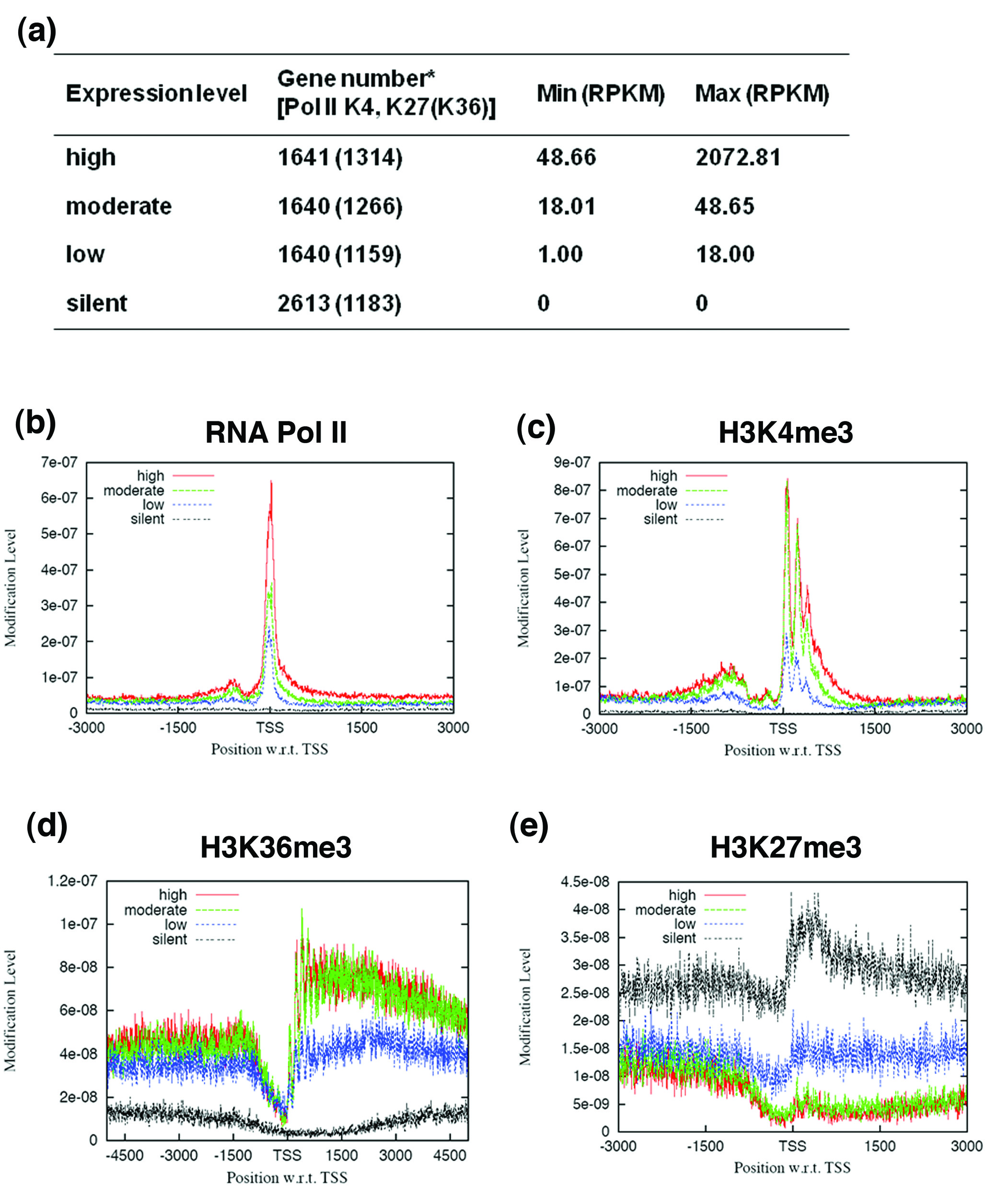

Supplement: Additional file 10 — Summary of the ChIP-seq results using Drosophila S2 cells. (a) The four groups of genes were classified according to their RPKM value based on the RNA-seq results. The numbers in brackets denote genes used for H3K36me3 (K36) analysis. *See Materials and methods for gene selection criteria. (b-e) Antibodies used for ChIP-seq are: (b) anti-RNA Pol II (Pol II); (c) anti-H3K4me3 (K4); (d) anti-H3K36me3 (K36); and (e) anti-H3K27me3 (K27). Enrichment of each histone modification and RNA Pol II is plotted over a -5 to +5-kb region with respect to the TSSs of the genes. [file gb-2010-11-4-r42-S10.jpeg]

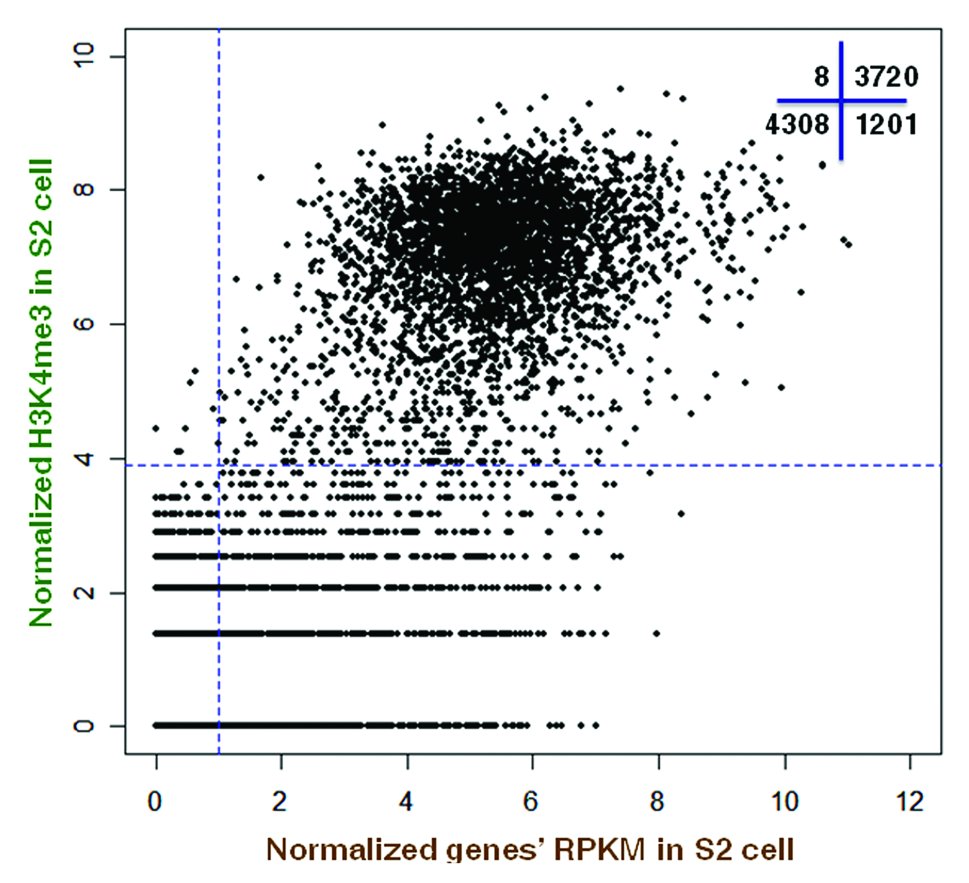

Supplement: Additional file 11 — Most genes enriched with H3K4me3 are actively expressed in S2 cells. Scatter plot of H3K4me3 enrichment and RPKM values for all annotated genes. All labels are the same as in Figure 2d, except RPKM = 1 was used as a cutoff for expressed genes. [file gb-2010-11-4-r42-S11.tiff]

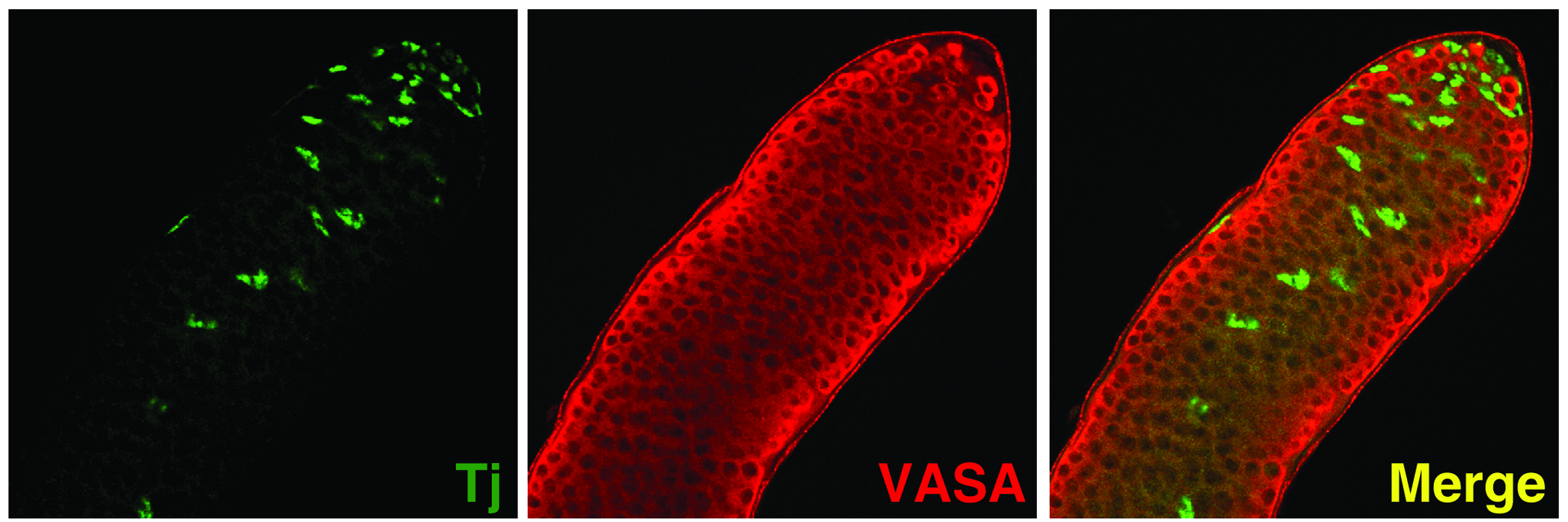

Supplement: Additional file 12 — Immunostaining of the bam testis with antibodies against the germ cell marker Vasa and the somatic marker Traffic jam (Tj). [file gb-2010-11-4-r42-S12.tiff]

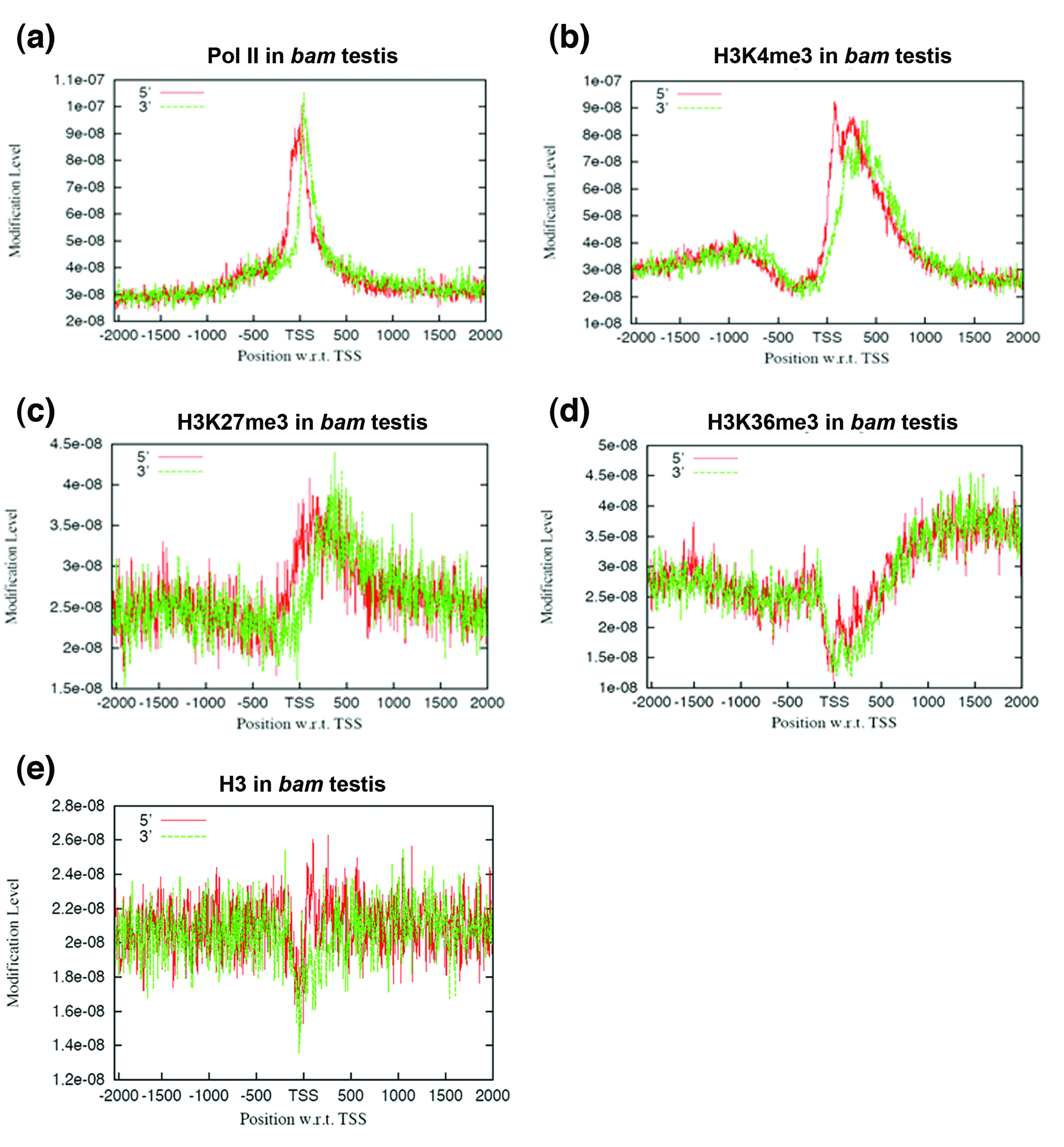

Supplement: Additional file 13 — Determination of the fragment size for ChIP-seq analysis, based on the distance between the 5' and 3' sequencing read peaks. All expressed genes (RPKM ≥ 1) were used for the modification level plots over a -2 to +2-kb window with respect to the TSS. (a-e) Antibodies used for ChIP-seq in bam testis were: (a) anti-RNA Pol II; (b) anti-H3K4me3; (c) anti-H3K36me3; (d) anti-H3K27me3; and (e) anti-H3. [file gb-2010-11-4-r42-S13.tiff]

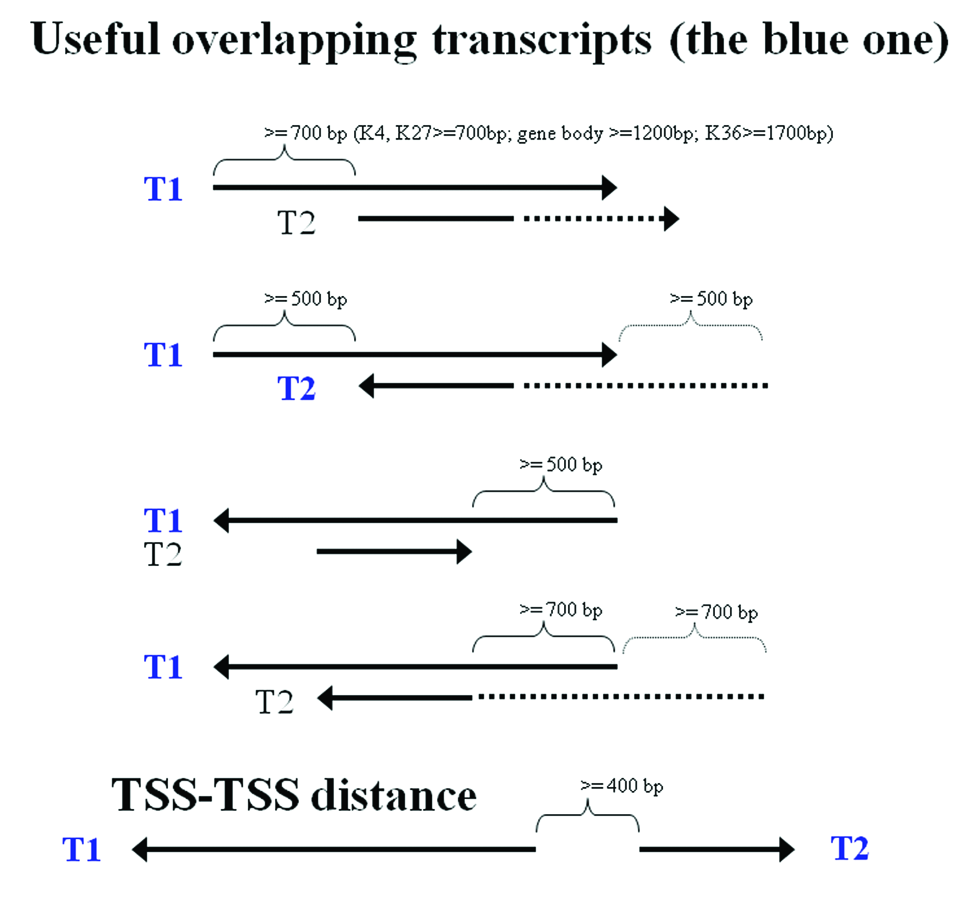

Supplement: Additional file 14 — Cartoons showing overlapping transcripts from different genes that were retained for data analysis. The transcripts labeled by blue letters were retained for data analysis, in each situation shown for overlapping genes. [file gb-2010-11-4-r42-S14.tiff]
